# Widespread ecosystem effects of an extreme fresh pulse event in a temperate marine ecosystem

**DOI:** 10.1038/s41598-026-57921-4

**Published:** 2026-06-28

**Authors:** Lesley H. Thorne, Janet A. Nye, Jacqueline M. McSweeney, Joseph Warren, Roy Price, Nathan Hirtle, Joshua Meza-Fidalgo, Tyler Menz, Toniann Keiling, Katherine Gallagher, Chelsi Napoli, Zachary Hoffman, Julia Stepanuk, Hannah Blair, Timothy V.N. Cole, Arthur Kopelman, Marianne McNamara, Orla O’Brien, Jessica Redfern, Rachel Carlowicz Lee, Paige Tortorice, Monique Escalante, Alison Ogilvie, Haocheng Yang, Lang Ming

**Affiliations:** 1 School of Marine and Atmospheric Sciences, Stony Brook University, Stony Brook, NY 11794-5000 USA; 2 Department of Earth, Marine and Environmental Sciences, Institute of Marine Sciences, Institute of Marine Sciences, The University of North Carolina at Chapel Hill, Morehead City, NC 28557 USA 3431 Arendell St,; 3 Department of Ecology and Evolution, Stony Brook University, Stony Brook, NY 11790 USA; 4 Bigelow Laboratory for Ocean Sciences, 60 Bigelow Drive, East Boothbay, ME 04544 USA; 5 Northeast Fisheries Science Center, National Marine Fisheries Service, National Oceanic and Atmospheric Administration, Woods Hole, MA 02543 USA; 6 Coastal Research and Education Society of Long Island, Inc, PO Box 54, West Sayville, NY 11796 USA; 7 Anderson Cabot Center for Ocean Life at the New England Aquarium, Boston, MA 02110 USA; 8 IBSS Corporation in Support of NOAA Fisheries, Silver Spring, United States

**Keywords:** Extreme event, Salinity, Right whale, Humpback whale, *Calanus finmarchicus*, Aragonite, Climate sciences, Ecology, Ecology, Environmental sciences, Ocean sciences

## Abstract

Extreme climate events are becoming more frequent and intense under climate change, but their impacts on marine food webs are not well understood. In the North Atlantic, salinity anomalies over decadal timescales have been well studied, but extreme freshening has been observed in recent years at a magnitude far greater than prior measurements. Further, short-term fresh pulses and their ecological effects are poorly understood. We integrate recent monitoring data from the New York Bight with long-term datasets to document the ecosystem impacts of a short-term fresh pulse which influenced this region from September 2023 to September 2024. Mean surface salinity in this region was lower from February through August than in any other year of observation in a 30-year dataset, with salinity anomalies of -0.80 to -1.43 psu (mean − 1.13 psu). Profound ecosystem impacts of this event were observed throughout the food web. We observed aragonite saturation levels below 1, reflecting corrosive conditions for calcifying organisms. Elevated densities of key copepods occurred in spring and summer, while fish and invertebrates showed marked equatorward shifts in a reversal of recent poleward trends. Humpback whale body condition and residence time were unusually high. Anomalously high sightings rates of endangered North Atlantic right and sei whales occurred during a particularly fresh period in summer. Our findings address a gap in knowledge regarding the ecological impacts of fresh pulses, and portend widespread impacts of these events under continued climate change with implications for commercial and protected species.

**Supplementary Information:**

The online version contains supplementary material available at 10.1038/s41598-026-57921-4.

## Introduction

Extreme climate events are becoming increasingly common^1–3^, a trend which is projected to continue under continued greenhouse gas emissions^4–7^. In marine ecosystems, heatwaves have become more frequent, longer, and more intense in recent decades^8–12^, with profound ecological and socioeconomic impacts^13–17^. These events can lead to species distribution shifts, mass mortalities, changes to food web dynamics, loss of fisheries income, and erosion of essential ecosystem services^16^. A single marine heatwave can result in direct economic losses of more than $800 million^16,18^.

While marine heatwaves and their effects on marine organisms have been studied in detail in recent years, the ecological impacts of other extreme events, such as those associated with low oxygen concentrations, high acidity, or low salinity, are often not well understood^19–22^. Extreme freshening events known as Great Salinity Anomalies have been observed over decadal time scales in the North Atlantic since at least the 1970s^23,24^. However, a recent extreme freshening event in the Northeast Atlantic from 2012 to 2016 was the largest and most rapid freshening event over the past 120 years^25^, and highlights the need to better understand the effects of extreme freshening events on marine organisms and food webs. Further, extreme events are typically studied retroactively, with the effects and implications of these events typically realized long after their occurrence^26^. However, observing and assessing these events as they are occurring, or soon thereafter, is critical to implementing effective climate adaptation strategies for coastal socioecological systems.

**Fig. 1:**
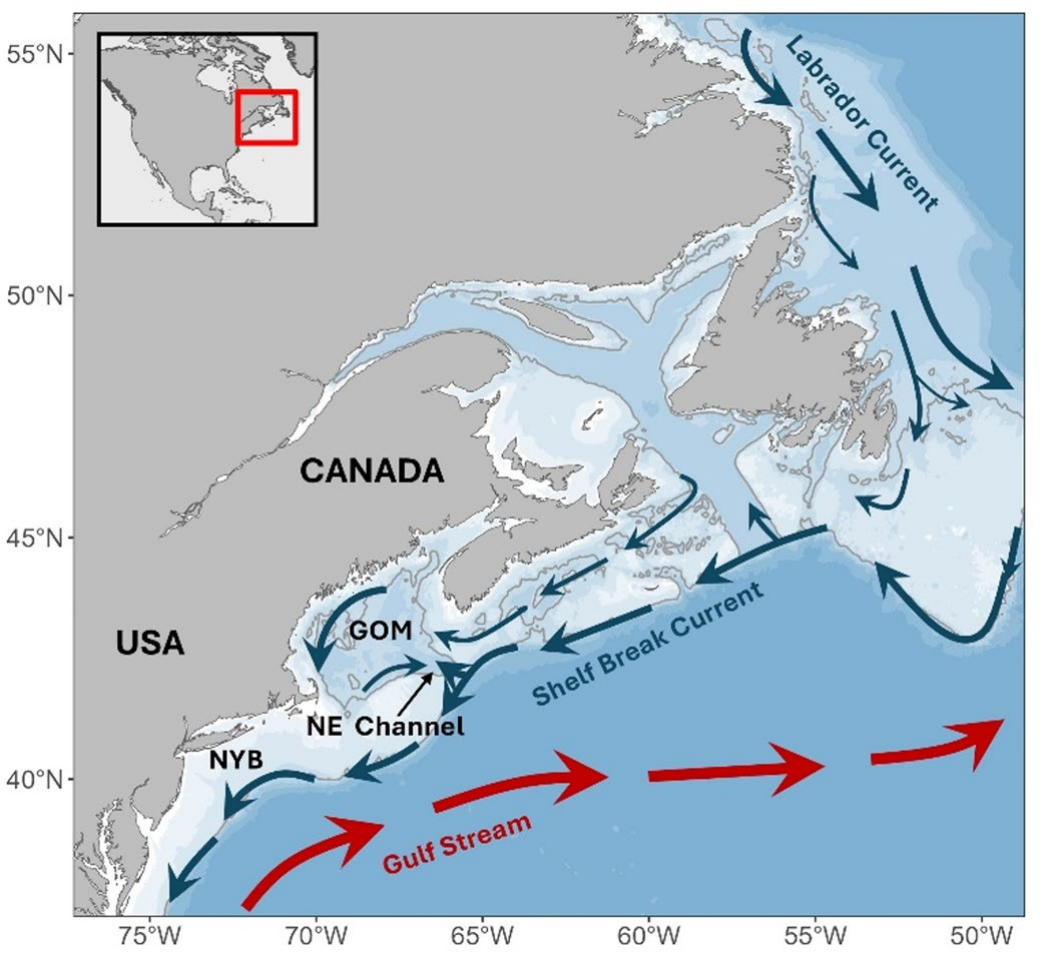
Regional circulation in the Northwest Atlantic continental shelf region (following^156–159^ and references therein). The study area on the New York Bight (NYB) shelf occurs downstream of the Gulf of Maine (GOM).

The Northeast United States (NEUS) has experienced rapid warming in recent decades, leading to widespread effects on marine organisms and fisheries^27–30^. Oceanographic observations since 2011 suggest a change in the source water entering the Gulf of Maine, from cold, fresh water from the Labrador Sea prior (Fig. 1) to 2011 to warmer, saltier slope water associated with the Gulf Stream from 2011 to 2023 following shifts in the Gulf Stream^26,31^. However, the onset of a “cold wave” was observed in late 2023 : a rapid shift to colder, fresher waters in the Gulf of Maine, likely due to a change in the source of water back to Labrador slope waters^26^. Oceanographic changes in the NEUS have strongly influenced the abundance and distribution of a variety of protected and commercially important species^29,32–35^. Long-term changes in the copepod community, including declines in prevalent and energy-rich copepods *Calanus finmarchicus*, have been observed in the NEUS in recent decades^35,36^. Many fish species have shown poleward shifts in distribution in association with warming waters^29,37^, while fish assemblages have shifted towards warm water species^32^. The Mid-Atlantic Cold Pool, a cold and fresh water mass occurring during spring and summer over the mid and outer continental shelf that is a remnant of winter mixing, is warming and shrinking^38^, with implications for the recruitment and distribution of cold water fish species^39^. Odontocete distributions have shifted poleward^34,40,41^, and critically endangered North Atlantic right whales (*Eubalaena glacialis*) have abandoned traditional foraging grounds in the eastern Gulf of Maine and B^43^ay of Fundy in association with declines in *C. finmarchicus*, their primary prey^30,42,^.

Recent studies of the impacts of climate change on marine organisms in the NEUS have largely focused on warming. However, cold, fresh events and changes in stratification strongly impact the plankton community including changes in the abundance of key copepod species *C. finmarchicus*, which in the NEUS are associated with cold, low-salinity water^44,45^. Cold, fresh events can also influence calcifying biota^46,47^; colder and fresher seawater generally contains lower concentrations of carbonate ions, which are required by many marine plankton and invertebrate species to build and maintain their shells or coral skeletons. These key changes highlight the need to better understand the ecosystem effects and spatiotemporal extent of the cold, fresh event observed in the Gulf of Maine in late 2023 and 2024^26^. Ongoing oceanographic and ecological monitoring in the New York Bight (NYB) within the NEUS allows us to document the response of multiple trophic levels to this fresh pulse in detail.

**Fig. 2:**
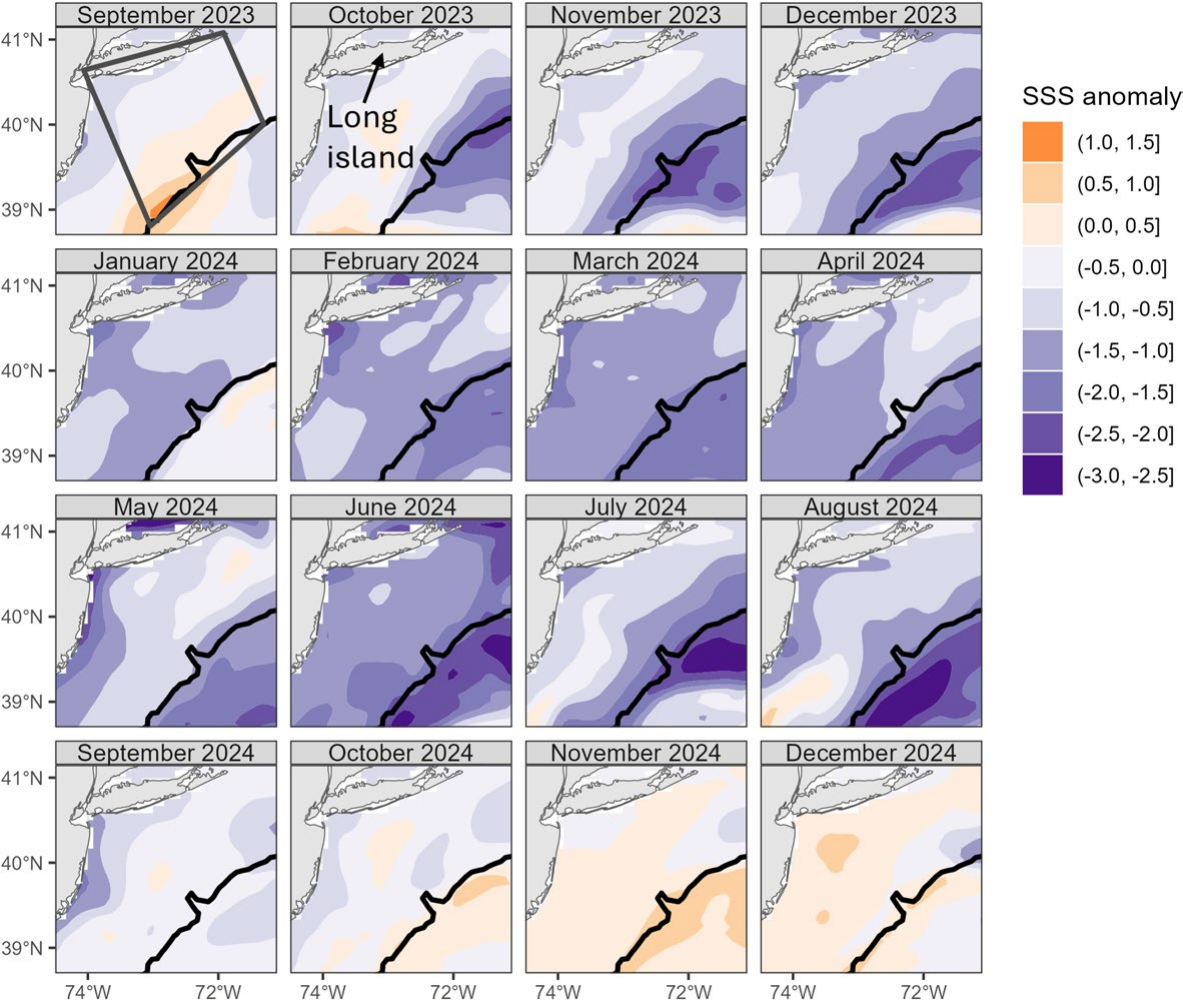
Monthly sea surface salinity (SSS) anomaly from September 2023 through December 2024. Surface salinity anomaly values were calculated from the 1993–2023 monthly means. The extent of the New York Bight study area is shown in the top left panel in dark grey. The 200 m isobath, representing the continental shelf break, is shown in black in all panels.

The NYB is a region in the NEUS south of the Gulf of Maine which extends from the southern coast of Long Island to the continental shelf break (Figs. 1 and 2). Waters of the NYB provide habitat for fishes that support recreational and commercial fisheries^48–55^ and serve as migratory and foraging habitat for a number of large whale species^56^. Humpback whales (*Megaptera novaeangliae*) feed on fish and invertebrates in the NYB during summer months^57–59^, while endangered North Atlantic right whales and sei whales (*Balaenoptera borealis*) feed on copepods and are typically observed in winter and spring months^56,60,61^. Since 2018, an ecosystem monitoring program has provided seasonal assessments of key ocean indicators in the NYB, encompassing physical oceanographic processes and lower, mid, and upper trophic levels using boat-based surveys and ocean gliders^62^. Observations from this monitoring program document anomalous conditions in both physical and biological observations in spring and summer 2024 reflective of the cold wave described by Record et al. (2024). Here we integrate this local monitoring data with available data from longer term physical and biological sampling programs and products to assess this event and its impacts on the food web in the NYB. Specifically, we examine how the following metrics in 2024 compare to previous years: temperature, salinity and aragonite saturation state of waters in the NYB; the volume of the Cold Pool and the volume of low salinity water; the abundance and distribution of zooplankton and fish; the species composition and relative abundance of seabirds; the relative abundance of North Atlantic right whales and sei whales; and the occurrence, body condition and residency of humpback whales. Our overarching goal is to provide a timely assessment of this oceanographic anomaly to inform our understanding of how salinity anomaly events influence marine ecosystems, and the ecosystem implications of more frequent anomalous conditions.

## Results

**Fig. 3:**
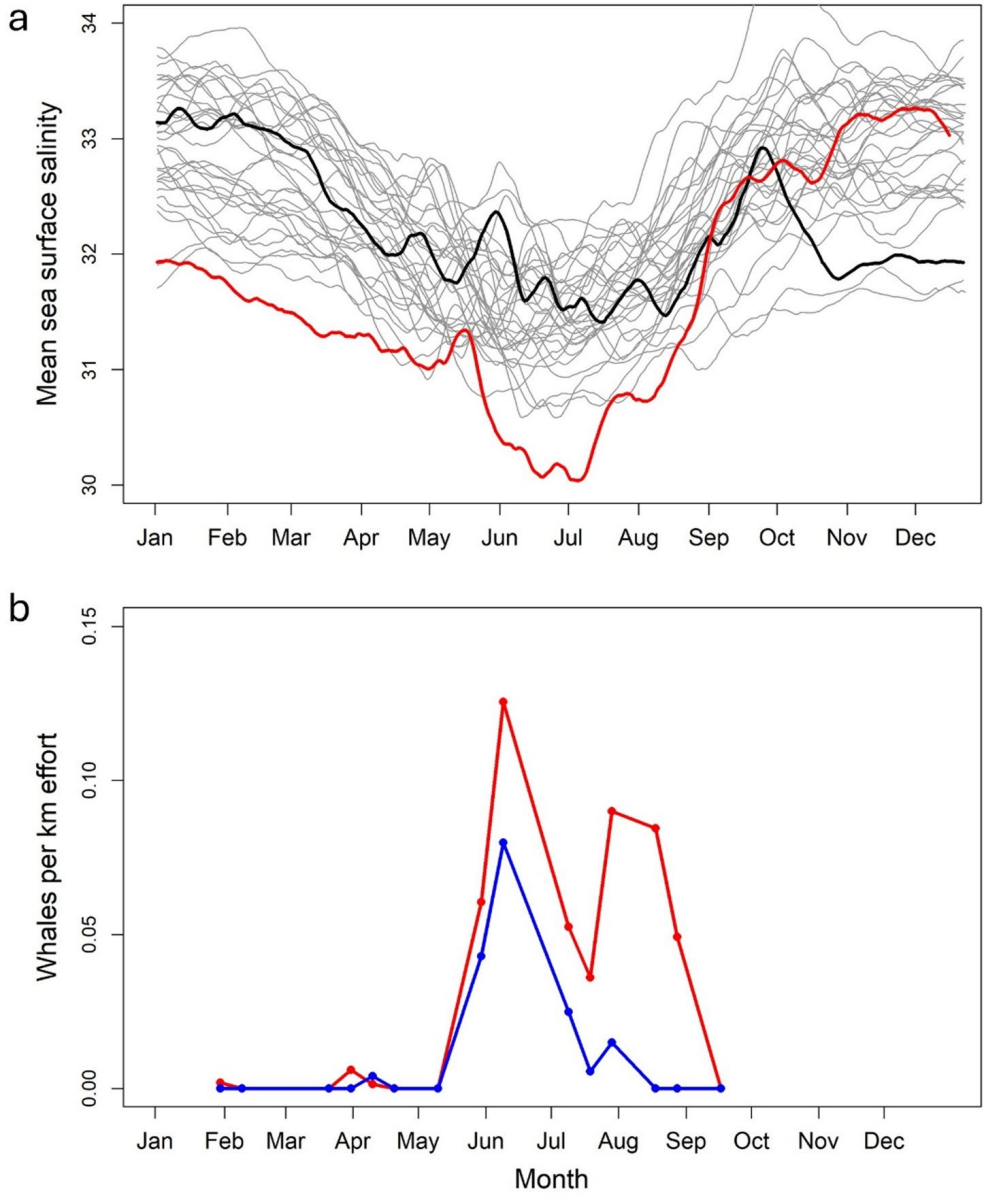
(**a**) Rolling ten day mean sea surface salinity for 2024 (red line) relative to 1993–2022 (grey lines) and 2023 (black line). (**b**) Ten day mean Sightings Per Unit Effort (SPUE) for North Atlantic right whales (red) and sei whales (blue) in 2024.

**Fig. 4:**
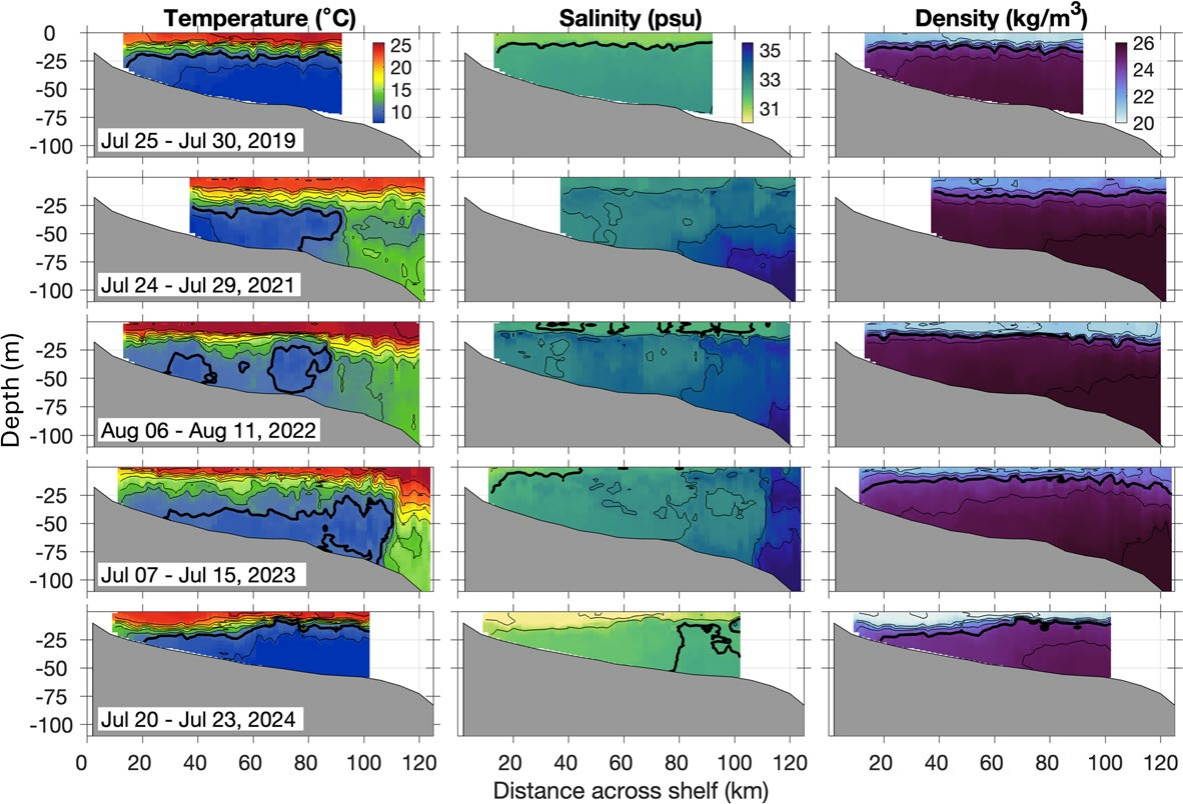
Cross-shelf glider transects of ocean temperature (2 °C intervals, 10 °C bold), salinity (1 psu intervals, 32 psu bold) and density anomaly (𝜌 -1000, 1 kg/m^3^ intervals, 24 kg/m^3^ bold) in the New York Bight during the summers of 2019, 2021, 2022, 2023 and 2024. Cross-shelf distance is measured from the nearest shore location. The glider transect in July 2024 occurred approximately 50 km west of glider transects in prior years.

**Fig. 5:**
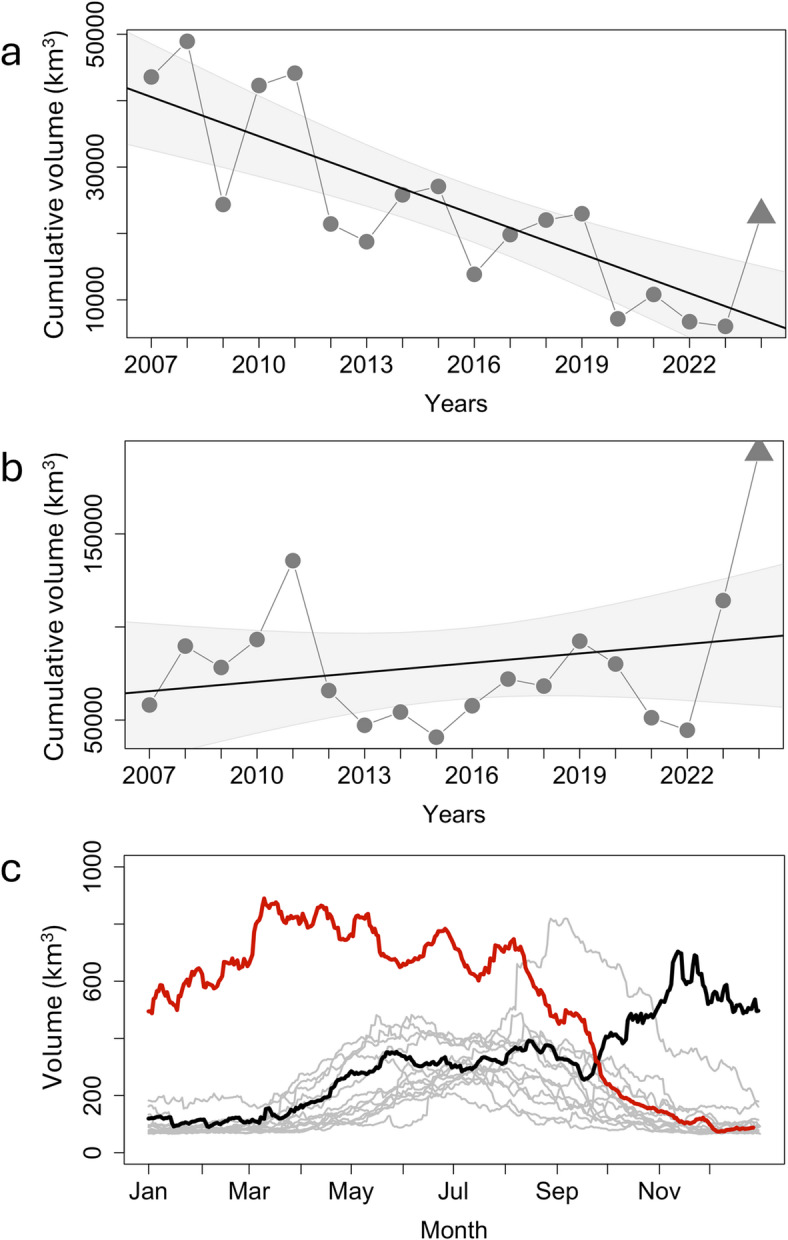
(**a**) Volume of the cold pool and (**b**) the volume of low salinity water (salinity less than 32 psu) by year from 2010–2024. (**c**) The seasonal progression of the volume of low salinity water is shown for the same years, with 2024 highlighted in red and 2023 in black. Gray lines represent 2010–2022. Data from Doppio, a ROMS-based (Regional Ocean Modeling System) model of the Mid-Atlantic Bight.

A pulse of cool, low salinity water began influencing the NYB ecosystem in fall 2023 (Figs. 2, 3, 4 and 5, Supplementary Fig. 1). The pulse was particularly evident in salinity conditions, with monthly mean surface salinity anomalies of -0.80 to -1.43 psu (mean − 1.10 psu) relative to the 1993–2023 mean from November 2023 through August 2024 (Fig. 2; Supplementary Table 1). From November 2023 through August 2024, monthly mean salinities were lower than the 5th percentile of 1993–2024 data for each respective month (Fig. 3), thus representing an extreme event^63^; in fact, the monthly means observed from February through August 2024 were the lowest monthly mean values in the dataset, which dated back to 1993. A period of particularly low salinity occurred from late May through July (Fig. 3a). Subsurface *in situ* glider data demonstrated that salinity in 2024 was anomalously low and that temperatures were cold but within the realm of typical interannual variability (Fig. 4, Supplementary Fig. 2). Both the glider and CTD data also corroborated the long-term analysis, with surface salinities of ~ 30 psu observed across nearly the whole shelf in July 2024 (Fig. 4, Supplementary Fig. 3), which is 1–2 psu fresher than surface in situ measurements from the last five years. Salinity conditions were particularly anomalous in summer as well as spring 2024 (Supplementary Fig. 2). In typical years, low salinity water (defined here as having a salinity value below 32 psu) is restricted to surface waters, but low salinity water was observed throughout the entire water column across much of the shelf in summer 2024 (Fig. 4). The annual mean volume of low salinity water in the NYB was higher in 2024 than in prior years (Fig. 5b, c). Negative sea surface temperature anomalies occurred in the NYB from October 2023 through February 2024 and again in May 2024 (Supplementary Fig. 1). While the volume of the Cold Pool showed a decreasing trend from 2010 to 2024, the volume in 2024 was considerably higher than in recent years (Fig. 5a). Freshwater inputs from rainfall, which could lead to higher freshwater riverine inputs from the coast within the year, did not explain the anomalously fresh conditions observed in 2024 (Supplementary Fig. 4), suggesting that the fresh anomaly is related to a change in the upstream shelf waters which advect down-shelf. The fresh pulse began to break down in mid-September 2024, approximately one year after its onset (Fig. 5c).

**Fig. 6:**
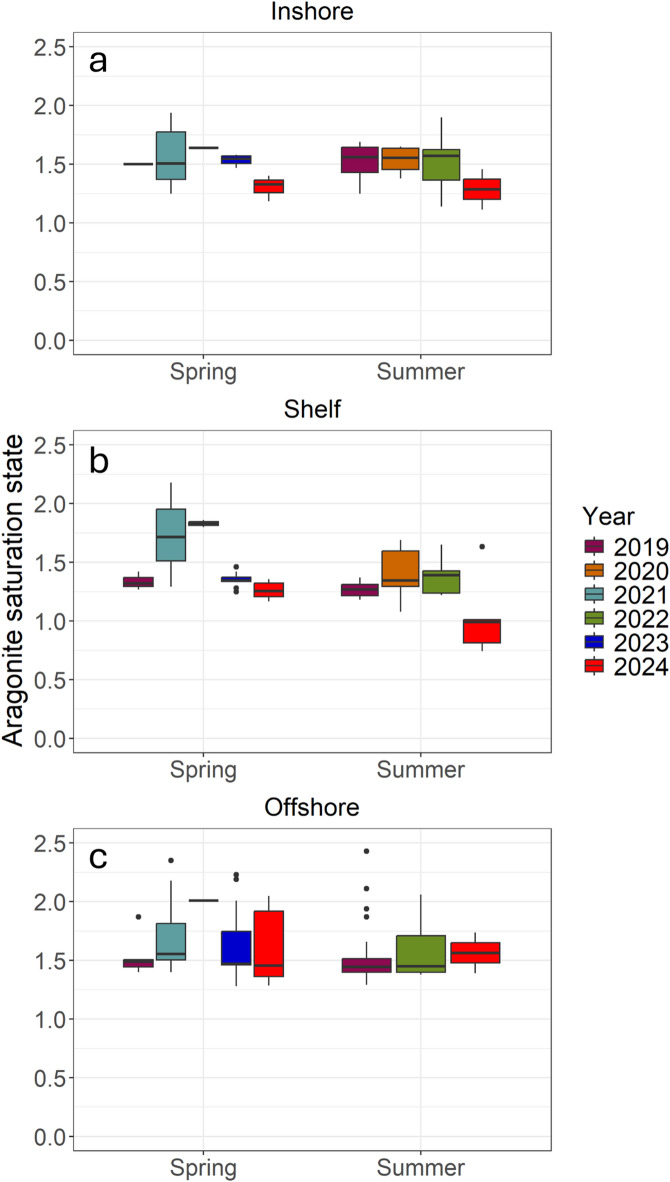
Aragonite saturation state in bottom waters in the New York Bight by year in (**a**) inshore, (**b**) shelf and (**c**) offshore regions for spring and summer, respectively, from 2019–2024.

For both aragonite saturation states and *Calanus finmarchicus* densities, we focus analyses on three distinct regions of the continental shelf: inshore, shelf, and offshore waters (see Methods). Aragonite saturation states in bottom waters of the NYB (i.e., within approximately 3 m of the seafloor) were significantly lower in shelf waters during spring 2024 in comparison to prior years (Fig. 6). The lowest aragonite saturation values observed in the 2018–2024 dataset occurred in summer 2024 in bottom water samples in shelf waters, with multiple observations below 1. While summer aragonite saturation states in bottom samples of shelf waters in 2024 were notably lower than in prior years (mean 1.04 in 2024 vs. 1.34 in 2019–2023; Fig. 6), the sample size for this region and season was small and the difference was not statistically significant after correcting for multiple comparisons (Supplementary Table 2). Spring surface water (samples obtained within approximately 2 m of the surface) aragonite saturation states in 2024 were statistically different than prior years for all three regions (inshore, shelf and offshore), with summer surface water values in 2024 not being significantly different from prior years in any of these regions (Supplementary Fig. 5, Supplementary Table 2).

**Fig. 7:**
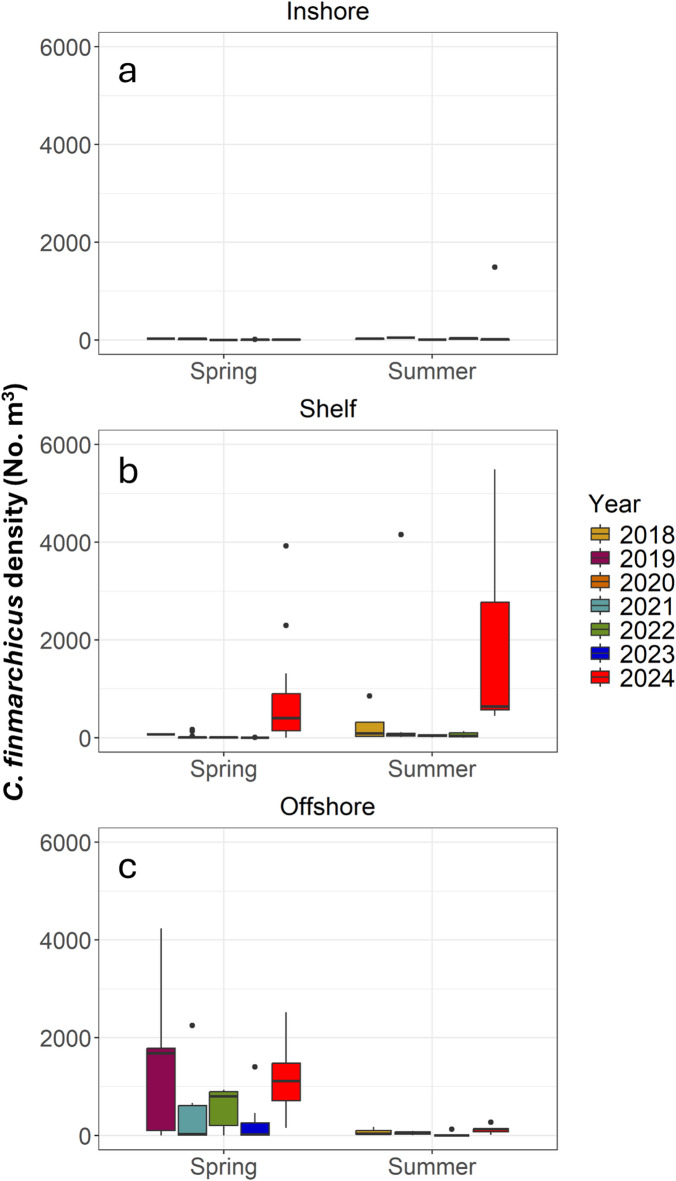
Density of *Calanus finmarchicus* (number per m^3^) in surface tows in the New York Bight by year in (**a**) inshore, (**b**) shelf and (**c**) offshore regions for spring and summer, respectively, from 2018–2024.

*C. finmarchicus* densities were significantly higher in 2024 in shelf waters than in prior years during both spring and summer (Fig. 7, Supplementary Table 2). Additionally, the distribution of *C. finmarchicus* in summer 2024 differed from typical across-shelf distributions. Long-term trends in the abundance and distribution of *C. finmarchicus* from Ecomon data showed that the species is generally most abundant in the NYB in offshore waters (Supplementary Fig. 6). In summer 2024, densities of *C. finmarchicus* in shelf waters were higher than those observed in all depth groupings and in any prior summer (Fig. 7). Although some tows in offshore waters in spring 2019 showed high *C. finmarchicus* densities, densities in this year were highly variable. In contrast, *C. finmarchicus* densities in offshore waters in spring 2024 were consistently high (Fig. 7c). As a result, when comparing 2024 to prior years, 2024 was found to have significantly higher *C. finmarchicus* densities in offshore waters in spring than prior years.

**Fig. 8:**
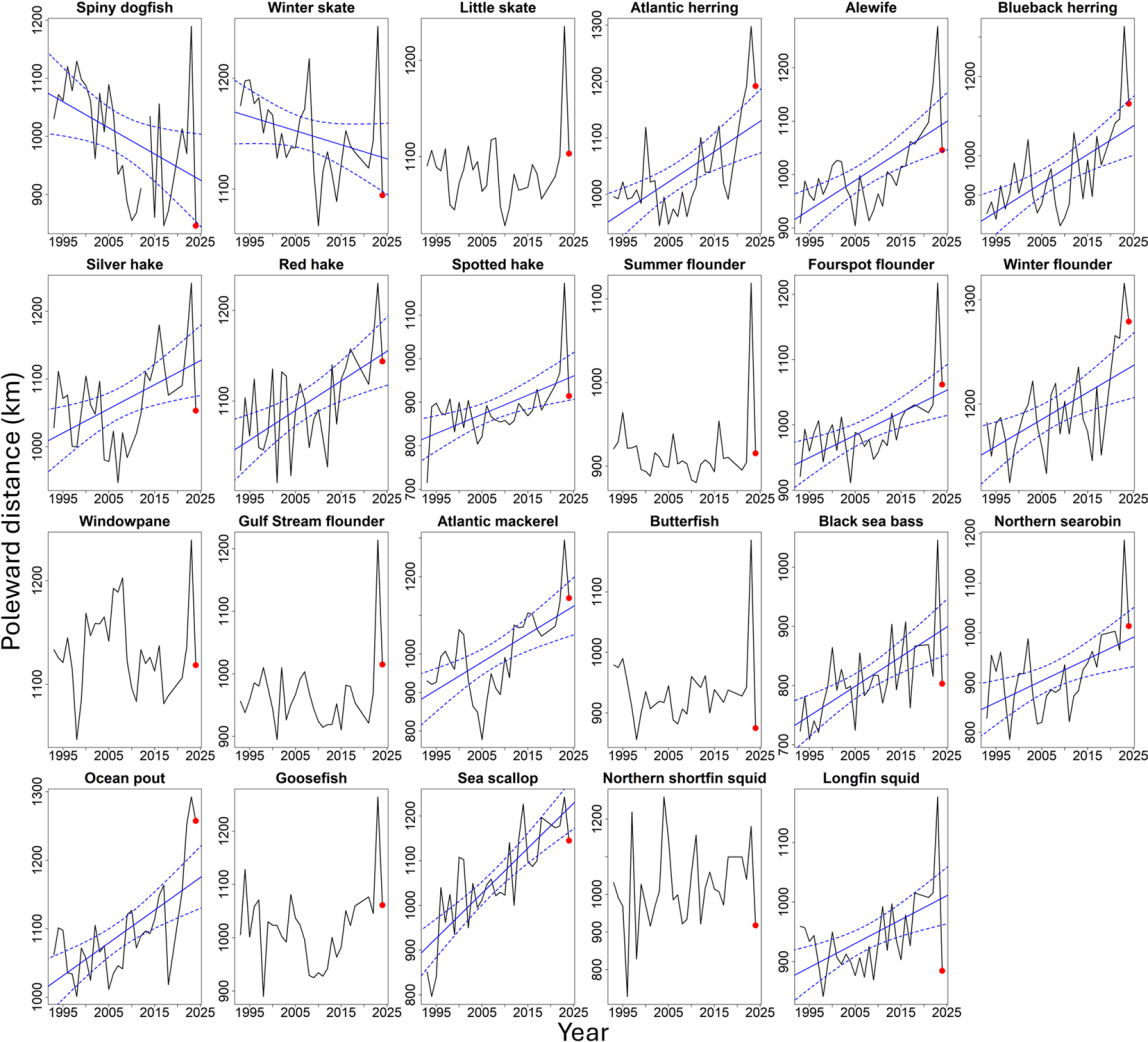
Annual changes in the poleward distance (distance from Cape Hatteras) of the center of biomass for fish and invertebrate species in the Northeast US. The red dot reflects data in 2024. Blue solid line represents trend from linear model (when significant at the *p* = 0.05 level), dashed blue lines represent confidence intervals.

All fish and invertebrate species examined showed equatorward shifts in their distribution in 2024 compared to previous years (Fig. 8). Of the 23 species assessed, 14 showed significant poleward shifts in distribution from 1993 to 2023, and their distributions in 2024 represented a reversal of this poleward trend. Two species showed significant equatorward shifts in their distribution from 1993 to 2023. All species showed an equatorward shift in distribution from 2023 to 2024. For 19 of the 23 species assessed (Supplementary Table 3), the equatorward shift from 2023 to 2024 represented the largest equatorward shift in the center of biomass observed since 1993. Equatorward shifts for ten species in 2024 were particularly marked relative to the prior distribution of these species with shifts of more than 200 km from 2023 to 2024.

Humpback whales showed higher residence time and estimated abundance in the eastern NYB in 2024 in comparison to 2018–2023 (Supplementary Fig. 7). Body condition of humpback whales was significantly higher in 2024 than in prior years (Supplementary Table 2). Relative abundance (whales per km survey effort) was higher for sei whales in May through July and for right whales from May through August than in prior years with available survey data back to 2000 (Supplementary Fig. 8). Relative abundance for both species was similar to that in prior years from January through April, and September through December. While more aerial survey effort focused around the Hudson Canyon in the southwestern region of the study area in 2024 than in prior years after right whales were observed in this region, relative abundance of right whales was considerably higher in 2024 even when excluding this region from analyses (Supplementary Fig. 9). The period of high right and sei whale relative abundance coincided with the period of very low salinity from late May through July (Fig. 3) and high densities of *C. finmarchicus* in shelf waters. During this time, right and sei whales were observed on the mid to outer shelf, regions where anomalously fresh shelf waters interacted with salty waters associated with Gulf Stream intrusions.

In winter 2023–2024 and spring and summer 2024, the relative abundance of seabirds was higher in comparison to observations in these seasons during other years (Supplementary Fig. 10), though this difference was only significant in summer after correcting for multiple comparisons (Supplementary Table 2). The relative abundance of seabirds in fall 2024 was similar to that in prior years. Fall 2024 seabird surveys were conducted in October, after the fresh pulse event had broken down.

## Discussion

**Fig. 9:**
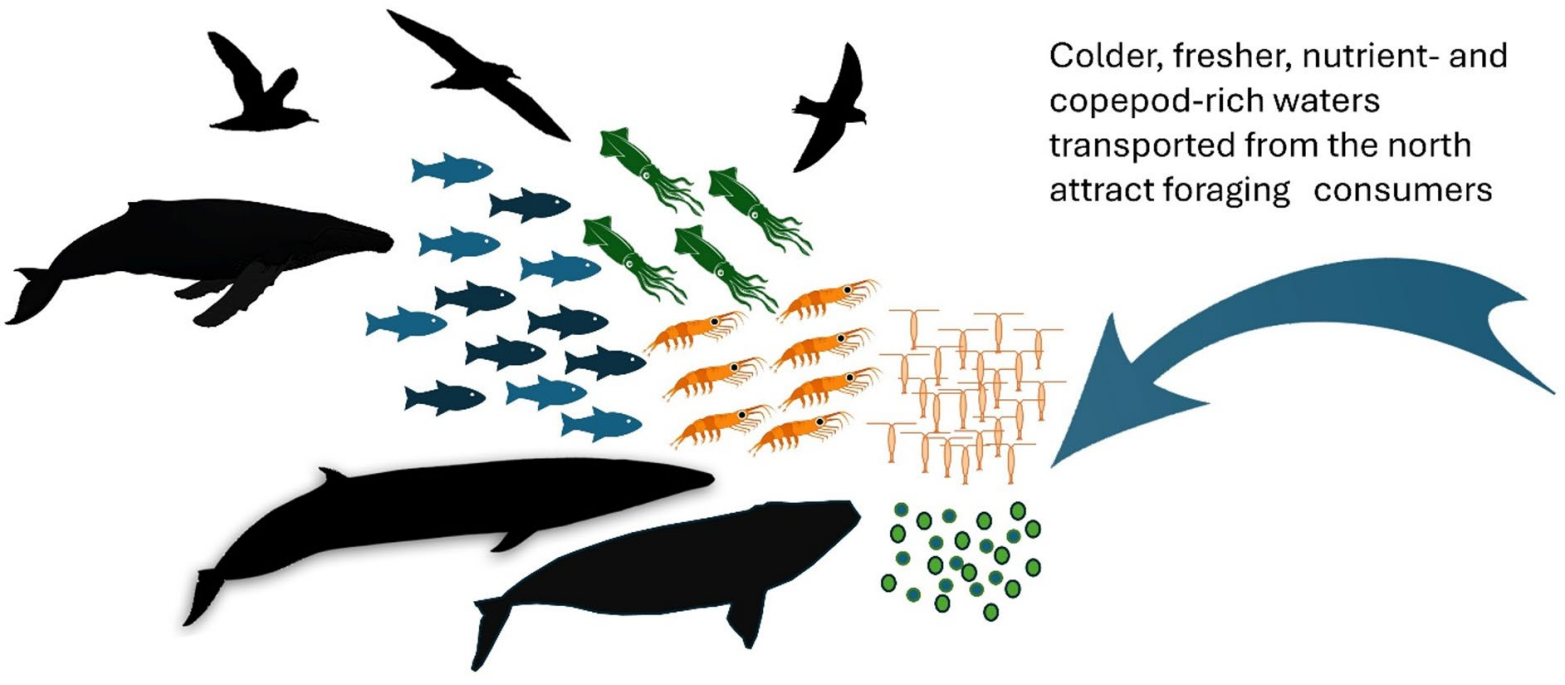
Conceptual diagram demonstrating the proposed mechanism through which the fresh pulse influenced marine organisms. Cold, fresh, nutrient- and copepod-rich waters from upstream created enhanced foraging opportunities for copepod consumers (fish, invertebrates, right whales, sei whales, seabirds such as storm petrels). Aggregations of mid-trophic level species then attracted large consumers such as North Atlantic right whales, which feed on copepods; sei whales which feed on copepods, larger invertebrates and fish; humpback whales, which feed on fish and invertebrates and fish; and seabirds such as shearwaters and gannets, which feed on invertebrates and fish.

We observed profound and rapid changes to invertebrates, fish and upper trophic level species in the NYB during a pulse of cool, low salinity water which influenced the region from approximately November 2023 through September 2024. Decadal-scale salinity anomalies in the North Atlantic have been well studied^23,24,64^, but fresh pulses and their ecological impacts have received less attention. Prior work from the Gulf of Maine focused on deep water observations and characterized this pulse as a cold wave^26^; our results suggest that this pulse was a water mass event in which a different pelagic habitat was advected from upstream, the physical properties of which are best tracked by salinity. In fact, in 2024, mean surface salinity was lower from February through August than in any other year of observations, using a dataset spanning more than 30 years. Our findings address a key gap in knowledge regarding the ecological impacts of this event. We found that this pulse strongly impacted aragonite saturation states in the NYB and influenced species across trophic levels, with marked changes throughout the food web occurring within months of its onset. Similarly, rapid changes in the marine ecosystem were observed following the major heatwave occurring in the Gulf of Maine in 2012. The 2012 heatwave led to major ecological changes such as shifts in species’ distributions and seasonal cycles, increases in warmer water species such as longfin squid and American lobster and declines in subarctic species, including cod stocks, *C. finmarchicus*, and northern shrimp^28,65,66^. The rapid biological responses that we observed were opposite in direction to those observed during intense warming; while the 2012 heatwave led to an acceleration of poleward species shifts^65^, we found equatorward shifts in multiple species during the  2024 fresh pulse that signal a reversal of recent poleward shifts. Our observations suggest that the ecosystem changes associatedwith the fresh pulse were primarily due to the transport of nutrient- and copepod-rich water from the north (Fig. 9).

The fresh pulse that we examine was first observed by Record et al. (2024) in the Gulf of Maine, who suggested that this event represented the return of Labrador Slope source water in early 2024 following a more than decade-long period of warm slope water associated with the Gulf Stream entering through deep waters in the Northeast Channel. However, it was not clear whether this would lead to a concomitant increase in *C. finmarchicus*, with subsequent effects on upper trophic level species such as right whales, given the complexity of local dynamics and the importance of both internal population growth and external supply driving populations of *C. finmarchicus* in the Gulf of Maine^26,45,67^. Our results suggest that the fresh pulse from the north supplied the NYB with high densities of *C. finmarchicus* and attracted foraging consumers into more southernly waters. It is unclear whether ecological impacts were as pronounced in the Gulf of Maine. We observed equatorward shifts of multiple fish and invertebrate species, in which species shifted their distribution into waters considerably further south of the Gulf of Maine than in recent years, and increased use of the NYB by baleen whale species that are typically more abundant in more northerly waters including the Gulf of Maine in summer months^35,68–70^. Future research on the biological responses of this fresh pulse in the Gulf of Maine relative to regions further south, such as the NYB, would provide a more thorough understanding of the ecological impacts of this event.

The Gulf of Maine has historically been a key foraging area for North Atlantic right whales due to the high densities of *C. finmarchicus* occurring in this region^71–73^. By 2015, right whales largely abandoned foraging habitat in the eastern Gulf of Maine following declines in *C. finmarchicus* ^30,35,36^, with a large portion of the population favoring summer foraging grounds in the Gulf of St. Lawrence ^35,74–76^. In addition, right whales have used waters in the NEUS south of the Gulf of Maine more frequently since 2010, particularly during winter and spring months^35,60,77,78^. While the Nantucket Shoals region northeast of the NYB now provides important right whale foraging habitat^77,78^, right whales are observed more sporadically further south in the NYB^56,79^. The majority of research on right whale foraging behavior and associations with prey have been conducted in the Gulf of Maine, and much less is known about right whale foraging behavior in more southerly waters^36^. Sei whale distribution and habitat use are poorly understood^69^, and a lack of knowledge limits our understanding of their populations and status^69,80^. We observed high densities of *C. finmarchicus* in proximity to right and sei whale sightings in the NYB, suggesting that right and sei whale were foraging in this region. In summer months, densities of *C. finmarchicus* in shelf waters were frequently (40% of samples) over the hypothesized feeding threshold for right whales of 1000 copepods m^− 3^^81^.

Our results suggest that the fresh pulse stemmed from changes in waters north and upstream from the NYB (Fig. 1) which advected *C. finmarchicus* copepods into waters of the NYB. The relative abundance of right and sei whales and *C. finmarchicus* densities were greatest during a period of particularly low salinity water in the NYB in summer 2024 from June through August. Typically, right whales are observed occasionally in the NYB in winter and spring, while sei whales are rarely observed in this region^56^. The anomalously high observations of whales and copepods in summer months may have reflected the peak of the fresh pulse event in the NYB (lowest salinity values), and peak densities of copepods advected into the NYB. The observed link between right and sei whale foraging and the 2024 fresh pulse suggests that pulse events can directly impact the foraging habitat used by these species, creating novel habitat in summer months when the species are not typically observed in this region.

Documented changes in humpback whale occurrence and body condition, as well as the species and relative abundance of seabirds, demonstrate that the fresh pulse influenced multiple consumer species, including species that forage on larger prey items. Humpback whales are generalist feeders, feeding on invertebrates and schooling fish, and typically feed on sand lance (family *Ammodytidae*) and menhaden (*Brevoortia tyrannus*) in the NYB ^58,82,83^. The seabird species observed in the NYB in spring and summer 2024 feed on a range of fish and invertebrates. Our analyses of fish and invertebrates targeted by commercial and recreational fisheries in the NYB demonstrate effects on the distribution of mid-trophic level species broadly, while our observations of humpback whales and seabirds suggest that the anomalous conditions observed in 2024 created enhanced foraging opportunities in the NYB specifically. The NYB is the southernmost region along the US east coast where humpback whales forage regularly in summer, and while there is exchange between the NYB and the Gulf of Maine^84^, prior research found that humpbacks in the NYB have significantly lower body condition than those foraging in the Gulf of Maine^85^. The increase in the estimated number of humpback whales in the eastern NYB in 2024 suggests that some humpback whales that typically forage in the Gulf of Maine may have instead focused their foraging efforts, or spent more time than usual foraging, in the NYB.

Our findings highlight the potential for fresh pulses to disrupt traditional biogeographic patterns of protected species, with implications for interactions with anthropogenic threats. Currently, protections for critically endangered North Atlantic right whales rely on protected areas that are fixed in space and time and are based on historical habitat use of the species^35^. After right whales were found foraging regularly in the Gulf of St. Lawrence in 2015, a mass mortality event was observed due to entanglement in fishing gear and vessel strikes in a region lacking protective policies^35,74,86^. More than 3% of the population was lost as a result of this mismatch between habitat use and protections^35^. Our findings demonstrate that marked oceanographic events such as fresh pulses have the potential to further disrupt traditional patterns of habitat use, causing protected species such as right whales to occur in high numbers in regions lacking protections. For example, the Port of New York and New Jersey is the busiest port on the US east coast and increases in vessel traffic in this region have been linked with increases in large whale mortality^87^. Under vessel speed regulations, large vessels (65 feet and greater in length) are required to travel at 10 knots or less in an area extending 20 nautical miles from the Port of New York and New Jersey between November 1st and April 30th each year to reduce vessel strikes of right whales (50 CFR 224.105). These vessel speed regulations are based on traditional patterns of habitat use, wherein right whales were primarily observed in the NYB region in winter and spring months. The high relative abundance of right whales, as well as endangered sei whales, in summer months of 2024 demonstrates how extreme oceanographic events can put these species at risk by increasing habitat use in a region of dense shipping traffic at a time when federal protections are not in place. Dynamic management tools have been proposed and developed to identify and forecast regions of risk for large whales^88–91^, and the sightings of right whales in the NYB in July 2024 triggered multiple Dynamic Management Areas, in which mariners are requested to avoid or travel slowly to avoid collisions with whales. Our results highlight the utility and need for such management efforts given the dynamic nature of large whale habitat use in the face of rapid environmental change.

In addition to impacts on protected species, both long-term climate-driven changes and extreme climate events can influence marine fisheries by redistributing target species ^92–96^. Fisheries can adapt to climate-driven changes in the abundance and distribution of target species by changing where they fish or what they fish for ^97^. In the US, fisheries vary substantially in their sensitivity to climate change and their ability to adapt to changes^98,99^. Fisheries shift more slowly than their target species, and regulatory or economic constraints may limit the extent to which fisheries can adapt to climate-driven changes in target species^100^. Our results highlight the potential for short-term salinity anomalies to disrupt recent poleward trends in commercially important fish species in the NEUS. Multiple species, including Alewife (*Alosa pseudoharengus*), silver hake (*Merluccius bilinearis*), spotted hake (*Urophycis regia*), black sea bass (*Centropristis striata*), butterfish (*Peprilus triacanthus*), and longfin squid (*Doryteuthis pealeii*), showed equatorward shifts in the center of distribution of approximately 200 km since the previous year. However, the center of biomass did not shift these species as far south as their historical distribution, highlighting that this short-term event does not reverse the multidecadal poleward shifts in nekton distribution^37,101,102^. Short-term events with rapid changes in target species distribution such as this may be particularly difficult to respond to. While data from NOAA fisheries Commercial Fisheries Landings database for 2024 was not available at the time of submission for this paper, future studies could assess spatial changes in landings in 2024 in comparison to prior years.

While we observed positive impacts of the fresh pulse on *C. finmarchicus* and large whales in the NYB, carbonate chemistry analyses demonstrate that the fresh pulse was associated with aragonite-undersaturated water which negatively impacts calcifying organisms. A low aragonite saturation state can cause dissolution of carbonate, posing significant problems for organisms that form calcium carbonate shells and skeletons^103,104^. The impacts of reduced aragonite on calcifying organisms in the NYB is unclear at this time. Affected organisms could include pelagic organisms such as pteropods, and benthic organisms including oysters, clams, sea urchins, and corals, while internal structures such as otoliths and statoliths in fish and invertebrates can also be impacted ^103,105,106^. Responses to low aragonite saturation are species-specific^107,108^, so there is no specific cutoff or “threshold” value that can be applied across an entire ecosystem. For example, for pteropods, mild shell dissolution is observed at aragonite saturation states of 1.5, which is considered an early warning response, while survival is impacted at aragonite saturation states of ~ 0.95. Aragonite saturation states are thus considered to provide a rigorous basis for vulnerability assessments^109^. Our data indicate that aragonite saturation states were lower than normal during the pulse of cool, anomalously fresh water, with values reaching less than 1.5 consistently in summer 2024 surface and bottom water samples, including values < 1 in bottom waters. Waters of the mid-shelf seem to have been particularly affected by the cool, fresh anomaly. Further research is needed to assess how low aragonite saturation states such as those associated with the fresh pulse, and how the duration of future events, might impact calcifying organisms in this region.

The source of the 2024 cool, fresh pulse is not currently clear^110–115^. However, our findings highlight the widespread ecosystem impacts that would follow increases of low salinity water into the NE US, with major socioeconomic implications due to shifts in the abundance, distribution and, potentially, survival of commercially important and protected marine organisms. The accelerated melting of Arctic ice^116–119^ suggests that marine ecosystems in eastern Canada and the NE US are likely to be increasingly impacted by fresh events in the coming years. This work highlights the need to quantify the effects fresh events on marine foodwebs in order to anticipate the ecological and socioeconomic impacts of future events.

Together, our results demonstrate marked impacts of the 2024 cold, fresh pulse on the NYB ecosystem, with impacts on lower-, mid-, and upper-trophic level species, as well as both commercially important and protected species. This work highlights the potential for widespread ecological impacts of future fresh pulse events with implications for commercial and protected species, and exemplify the importance of in situ data collections for detecting and assessing ecological impacts of extreme events.

## Methods

### Long-term temperature and salinity trends in the New York Bight

To examine temperature and salinity trends from 1993 to 2024, we used NOAA OISST sea surface temperature data at a ¼ degree resolution (https://www.ncei.noaa.gov/products/optimum-interpolation-sst ), and sea surface salinity using daily Level-4 sea surface salinity data at 1/8 degree resolution from the E.U. Copernicus Marine Service Information, which combines data from multiple satellite sources with in situ salinity measurements (10.48670/moi-00051). We calculated monthly temperature and salinity anomalies from September 2023 through December 2024 from the 1993–2023 monthly means and assessed spatial trends in monthly anomalies to examine the onset and spatiotemporal extent of anomalous conditions during 2024.

To examine variability in salinity and temperature throughout the water column, we used data from Doppio, a ROMS-based (Regional Ocean Modeling System) model of the Mid-Atlantic Bight which assimilates data from satellites and in situ sampling^120,121^. Doppio is a depth-following model, dividing the water column into the same number of depth groupings across all water column depths, thus allowing for more detailed resolution of oceanographic trends at depth in coastal regions than global models, which provide data at a consistent depth resolution across all water depths. We examined trends in the volume of the Cold Pool from 2007 (the earliest year Doppio data were available) to 2024. The Cold Pool was defined as waters below 10 °C, and was only considered present when vertical stratification (defined as the change in temperature from the surface to the bottom, divided by the depth of the water column^122^) was greater than 0.2 °C per meter. We calculated the Cold Pool volume from DOPPIO data by multiplying the height of the Cold Pool within a grid cell by the area of the DOPPIO grid cell (7 × 7 km grid cells = 49 km2 area) and summing the volume across the NYB. Given the presence of anomalously fresh water observed in the NYB in 2024, we quantified the volume of low salinity water, defined as water below 32 psu, using the same approach used to assess Cold Pool volume. We calculated the cumulative volume of the Cold Pool and the cumulative volume of low salinity water in each year, respectively, by summing the volumes for each day of the year. We examined patterns in the volume of low salinity water in 2024 in comparison to 2007–2024. Additionally, we examined whether freshwater inputs from rainfall could have influenced the salinity trends observed in 2024 by comparing the total annual precipitation at McArthur Airport on Long Island to the annual average regional surface salinity from Doppio.

### Ecological indicators

Carbonate chemistry analyses focus on aragonite saturation states (Ω), which provides a useful indicator of impacts of oceanographic changes on calcifying organisms^47^. Aragonite is one of two mineral forms of calcium carbonite, and is produced by many corals, pteropods and mollusks. Generally, when the saturation state of aragonite is above 1, seawater is said to be supersaturated with respect to aragonite and theoretically the formation of shell structures is more favorable. When the aragonite saturation state is less than 1, seawater is said to be undersaturated with respect to aragonite^123^. Biological responses to specific aragonite saturation states vary by species, though there is general consensus that when aragonite saturation states decrease to < 1.5, there is cause for concern for biological processes considering cumulative effects ^124^.

Zooplankton analyses focus on *C. finmarchicus* as it is the dominant copepod species on the Northwest Atlantic shelf in terms of biomass, it is the primary prey species of North Atlantic right whales and right whale occurrence is tightly linked to its density^71,72,125^, and because its distribution in the NEUS varies strongly with oceanographic change^42,126–128^. We examined changes in the center of distribution of fishes and macroinvertebrates on the Northeast shelf, focusing on species occurring in the NYB (described in more detail below) to provide a broader spatial assessment of shifts in mid trophic level species.

Analyses of upper trophic level species focus on three species of baleen whale: humpback whales, North Atlantic right whales, and sei whales. Baleen whales forage and accrue energy reserves in high-latitude waters, including the NEUS, from spring through fall and then migrate to low-latitude breeding grounds for the winter. Humpback whales and North Atlantic right whales have shown increases in the use of the NYB region since 2010. Humpback whales have been regularly using the NYB as foraging habitat in summer and fall months since 2011^57–59^, likely due to both increases in Atlantic menhaden in this region, and to expansion of foraging habitat from the Gulf of Maine as the population increases^57,87^. North Atlantic right whales have shown increasing use of regions within and adjacent to the NYB over the last decade during winter and spring, and in recent years have been observed frequently in summer and fall, though it is unclear the extent to which right whales are foraging in this region^35,60,77,78^. Less is known about sei whale ecology in this region, though data from recent aerial surveys suggests that they occur most frequently in the NYB in spring on the continental shelf and slope^56^.

### Detailed in situ data sampling in the New York Bight

*In situ* observations from the NYB ecosystem monitoring program initiated in 2018 (hereafter “NYB Ecosystem Monitoring Program”) were used to assess detailed trends in oceanographic and ecological data within the NYB, and include data from ocean glider deployments, seasonal shipboard monitoring cruises, and targeted small boat surveys of seabirds and large whales. Ocean gliders, autonomous underwater vehicles that are propelled through changes in buoyancy^129^ were used to examine temperature, salinity and density at depth along tracklines in the NYB seasonally. We focus on a cross-shelf transect from Southhampton NY to the outer-continental shelf  (our most consistently monitored glider line) in summer (Supplemental Fig. 11). Seasonal shipboard monitoring cruises were conducted aboard the R/V Seawolf from 2018 to 2024, and included CTD casts, water sampling for carbonate chemistry analyses, and zooplankton tows at fixed stations along survey transects that extend from the coastline of Long Island to the continental shelf break (Supplementary Fig. 11). Analyses of carbonate chemistry data and zooplankton examine spring and summer months since these were consistently sampled across years.

Carbonate chemistry and zooplankton sampling on New York Bight Ecosystem Monitoring cruises on the R/V Seawolf was initially (2018–2021) conducted on seven cross-shelf transects, but survey design was modified in 2022 and four cross-shelf transects were conducted from 2023 to 2024 (Supplementary Fig. 1). Thus, while all cruises provide cross-shelf sampling, data from 2023 on represent fewer stations at different along-shelf locations. Additionally, not all transects could be completed in each season and year due to inclement weather. For this reason, and because we were interested in changes in carbonate chemistry and zooplankton density across the shelf in this study, we analyzed carbonate chemistry and zooplankton data by depth grouping for spring and summer. The number of stations sampled by season and year is provided in Supplementary Tables 5 and 6. Seasons were defined as follows: Spring, March through May; summer, June through August; fall, September through November; winter, December through February.

Carbonate chemistry samples were collected at each station using a rosette with six 10 L Niskin bottles that sampled at different depths of the water column. Data evaluated here are from water samples taken at the surface (~ 2 m) and the bottom (~ 3 m from bottom). Samples were collected from the Niskin into 500 mL borosilicate glass bottles and preserved with 100 µL of saturated HgCl_2_. Each sample had a headspace of < 1% of bottle volume. Samples were sealed with vacuum grease and ground glass stoppers to create an airtight seal. Samples were stored in a cool, dark area after preservation until the cruise was completed at which point they were moved to cold storage at Stony Brook University until analysis.

Zooplankton tows were conducted with a 0.6 m diameter, 333-µm mesh ringnet at each station by lowering the net to a depth of 25 m (or to 2 m above the bottom at sites with depths between 20 and 25 m) and raising it to the surface. Net contents were sieved and preserved in a solution of 10% buffered formalin and 90% seawater.

We examine annual variability in large whale body condition in the NYB as a metric of energy transfer to upper trophic levels, focusing on humpback whales which have been observed foraging regularly in New York waters in recent years and occur in accessible nearshore habitats^57,58^. Like other baleen whales, humpback whales are capital breeders that accumulate energy reserves while on foraging grounds^130–132^. Body condition of baleen whales can be assessed using morphometric measurements from Unoccupied Aerial Systems (UAS), or drones, which can inform the accumulation or depletion of energy reserves^133–135^. Additionally, we examine relative abundance of North Atlantic right and sei whales from aerial surveys to assess how the occurrence of these zooplankton foragers compares to variability in *C. finmarchicus* in the NYB.

Photo-identification studies and UAS assessments of body condition of humpback whales were conducted in the eastern NYB from 2018 to 2024. Photo-ID survey coverage was conducted by opportunistic small boat surveys from Stony Brook University and the Coastal Research and Education Society of Long Island (CRESLI) (Supplementary Fig. 11). We integrated photo-identification data for humpback whales from the NYB monitoring program with data from seasonal whale watching tours run by CRESLI from 2018 to 2024, when sufficient data from both programs were available to examine patterns of habitat use in humpback whales. Boat-based photographs of each humpback whale’s flukes and/or dorsal fins were used to photo-identify individual whales^136^ and to identify whales that were resighted.

We obtained UAS imagery of humpback whales for photogrammetric measurement using a DJI Phantom 4 Pro + UAS (2018–2022) or a DJI Mavic 3 Pro UAS (2023–2024) flown at an altitude of 15–40 m over humpback whales. This work was conducted under a National Marine Fisheries Service General Authorization (GA No. 21889) from 2018 to 2021, and Permit No. 26,260 from 2022 to 2024. UAS were flown by licensed Federal Aviation Administration (FAA) Part 107 pilots. The survey protocol for this work was approved by the Stony Brook University Institutional Animal Care and Use Committee. One of two methods were used to obtain accurate altitude measurements of UAS flights, which is key to obtaining accurate morphometric measurements for body condition analyses. For data obtained in 2018, we used the method of Burnett et al. (2019), and used CollatriX^137^ to correct UAS altitude estimates derived from the UAS barometer using measurements of an object of known size (the swim platform on our research vessel) taken at different heights. For data obtained thereafter, we used data from a SF11/c LiDAR laser altimeter installed on the UAS as in Dawson et al. (2017). The angle between the gimbaled camera and altimeter were accounted for as in Dawson et al. (2017).

We examined variability in sightings rates of seabirds in the NYB. Seabird survey effort in the NYB is low^138^ which limited our ability to understand the abundance, distribution and habitat use of pelagic birds in this region. Recent seabird surveys conducted seasonally in the NYB allowed us to assess seabird species in the NYB, and variability in use of the NYB relative to the fresh pulse event. However, seabird surveys in the NYB were initiated in summer 2022, and we were therefore able to assess differences in the relative abundance between years over a more limited time frame than for oceanographic, carbonate chemistry and copepod datasets. Surveys were conducted seasonally in the eastern NYB on four cross-shelf transects on the RV Paumanok from 2022 to spring 2025, which ran from inshore to mid-shelf waters (Supplementary Fig. 1). One observer on either side of the boat records seabird sightings as in^139^. In some seasons, not all transects could be completed due to inclement weather (Supplementary Table 4).

### Long-term ecological data

To provide a broader context for the ecological trends observed in the NYB Ecosystem Monitoring Program, we complemented these data with longer-term ecological datasets. Data from the North East Fisheries Science Center (NEFSC) Ecosystem Monitoring Program (Ecomon) surveys https://www.ncei.noaa.gov/access/metadata/landing-page/bin/iso?id=gov.noaa.nodc:0187513) from 2000 to 2021, the latest year available at the time that analyses were conducted, were used to assess long-term trends in *C. finmarchicus* and to provide broader context for trends in this species observed since 2018. Ecomon surveys are conducted six to seven times per year, and sample randomly selected stations in sub-regions within the NEUS during each survey. The Ecomon survey conducts 120 zooplankton tows at randomly selected stations in the NEUS each season, with approximately 68 tows per year in the NYB. Ecomon zooplankton tows are conducted using a 61 cm bongo net fitted with 333 μm mesh conducted for a minimum of five minutes and are run obliquely from within five m of the bottom or a depth of 200 m to the surface. Since the methodology for Ecomon tows differs from that use for zooplankton tows conducted in NYB ecosystem monitoring surveys, the two cannot be compared directly.

Data from the NEFSC spring trawl survey was used to examine changes in the distribution of key fish and invertebrate species. This survey uses a stratified random design and has occurred since 1968 (https://www.nafo.int/Portals/0/PDFs/sc/2014/ scr14- 024. pdf ). Details of the data collection for this survey are outlined in^140^.

We examined long-term variability in sightings of right and sei whales in the NYB (2001–2024) using aerial surveys of large whales conducted by the NEFSC and the New England Aquarium (NEAq) as part of NOAA’s North Atlantic right whale aerial survey effort. Both North Atlantic right whales and sei whales feed on dense aggregations of *C. finmarchicus* and occur in similar habitats^141,142^ and we therefore examined trends in both species. The habitat use of right whales often differs from that of other large whales that feed primarily on fish^143^, and since the right whale aerial surveys focus on habitats used by right whales, we did not use these survey data to examine habitat use of other large whales that occur in the NYB.

Aerial surveys used high-wing aircraft, consisting primarily of DeHavilland Twin Otters (NEFSC) and a Partenavia P68 (NEAq), flown at 750 or 1000 feet at 100 knots. Two observers, one on either side of the plane, and an additional data recorder (NEFSC) record effort and sightings as in^78,144^. Observers use a measure of overall quality of sighting conditions using sea state, cloud cover, glare intensity and visibility, and estimate visibility in nautical miles. Observers go off effort if visibility drops below two nautical miles or sighting conditions were recorded as poor^144^. The NEFSC has conducted the North Atlantic Right Whale Aerial Sighting Survey each year to locate and assess the distribution of right whales in the Northeast US. While the survey focuses on assessing the distribution of North Atlantic right whales and obtaining photo-identification images of right whales, sightings of other large whale species are recorded as well. Prior to 2017, the NEFSC surveys also recorded small cetaceans and large fish species. From 2001 to 2007, the NEFSC surveys included a stratified random survey scheme covering federal waters from New York to Maine. Starting in 2007, the surveys reduced the spatial coverage to right whale seasonal habitats, but in 2023 began filling in spatial coverage gaps from Maine to Virginia. While the survey scheme attempts to distribute effort in an unbiased manner, when aggregations of right whales are found, extra surveys are conducted focused on photographic mark recapture efforts – meaning survey effort varied in the NYB from year to year, though effort during photographic circling to confirm group size of sightings was not included in the effort calculation. Starting in 2024, NEAq contributed to these efforts by providing additional standardized survey effort in the Gulf of Maine, and as needed coverage of right whale aggregations in waters from New York to Maine.

### Analyses of aragonite saturation states and zooplankton density

Aragonite saturation state was analyzed from water samples at the Carbonate Chemistry Laboratory (CCL) at Stony Brook University’s School of Marine and Atmospheric Sciences from 2019to 2024 using community-accepted quality control protocols^145^ and certified reference materials (CRMs; for testing accuracy of DIC and TA) obtained from Andrew Dickson at UCSD Scripps Institution of Oceanography. Water samples in 2018 were run at a different lab with different quality standards, and we therefore excluded data from 2018 from analyses. Field duplicate samples were analyzed to evaluate reproducibility. DIC and TA were analyzed separately using an automated analyzer (VINDTA 3 C #089, Marianda) with a precision of ± 1 µmol kg-1 for both DIC and TA. Aragonite saturation state (Ω) was calculated from DIC and TA data using CO2SYS^146^ K1 and K2 dissociation constants were from^147^ refit as suggested by^148^. For aragonite calculations, in-situ temperature and salinity were used, along with the total pH scale and pressure from the CTD. To assess densities of *C. finmarchicus*, subsamples (containing > 100 individuals) were enumerated and identified to species, and used to estimate numerical densities (individuals m^− 3^).

We examined annual and seasonal patterns in aragonite saturation state and the abundance and distribution of *C. finmarchicus* within three distinct regions of the continental shelf to examine cross-shelf differences: less than 35 m, 35–70 m and greater than 70 m, referred to as inshore, shelf, and offshore waters, respectively. Water depths in the NYB increase gradually over the shelf until depths of approximately 100 m, and then drop off sharply (Supplementary Fig. 11). While samples were not evenly available in these three depth groupings (Supplementary Tables 5,6), we sought to separately examine aragonite saturation and *C. finmarchicus* in nearshore and more offshore waters given differences in prior observations of *C. finmarchicus* in these regions (Supplementary Fig. 6). Surveys in winter and fall were less consistent between years and we therefore focused on assessing data in spring and summer. For each region (inshore, shelf, and offshore) and season (spring and summer), respectively, we examined differences in aragonite saturation and *C. finmarchicus* density between 2024 and prior years using Wilcoxon tests, correcting for multiple comparisons using Bonferroni corrections (alpha value of 0.05/3 to account for comparisons in inshore, shelf, and offshore waters, respectively).

### Distributional shifts in fish and invertebrates

Using data from the NEFSC spring trawl survey, we examined changes in distribution of fishes and macroinvertebrates on the Northeast shelf since 1993 to be consistent with the time series available for surface salinity. We selected fish and invertebrate species that were present in at least 80% of years of trawl survey data since 1993, and present in at least 5 tows in each spring survey since 2000 in the NYB. For these species, we quantified the annual biomass-weighted mean location as in^37,40,101^ using only offshore strata^149^. Changes in latitude do not fully capture along-shelf distances in the NEUS due to the curvilinear shelf so we therefore examined changes in poleward distance, assessed as distance from Cape Hatteras, North Carolina as in^37,40^. We calculated distance from Cape Hatteras using the Spatial Analyst package in ArcGIS (version 10.8.1), with the coastline and the shelf break as barriers. We used linear regressions to examine annual changes in the biomass-weighted mean poleward distance for each species.

### Humpback whale photo-identification and body condition analyses

The humpback whale photo-ID catalogs from Stony Brook University and CRESLI were visually compared, utilizing both flukes and dorsal fins to determine any matches between them. At least two trained observers had to agree on a match for it to be confirmed. Matches between individual whales were used to create a list of sighting dates and locations for each individual whale from this combined dataset. We estimated the residence time and number of humpback whales in the eastern NYB as in^150^ by fitting a lagged identification rate model in the program SOCPROG^151^ (version 2.10) to photo-ID sightings. This approach calculates the probability of reidentifying an individual within the study area after a certain time lag via maximum likelihood.

To assess humpback whale body condition, aerial images were first evaluated for adequate quality on a scale of 1 to 3 according to criteria defined by^134^. Two analysts scored images; images with a score of 1 (good) or 2 (medium) for all categories were used for further analysis, while images with a score of 3 (poor) in any category were excluded from further analysis. We calculated the Body Condition Index (BCI) for humpback whales as the residual of the expected value from the log-log relationship between total length of total body volume^85,134,152^. BCI as a metric is standardized to total length, and thereby incorporates individual measurement uncertainty^85,134,152^. We calculated BCI using widths and total length measurements as in^85^. Briefly, we conducted length and width measurements in MorphoMetriX^153^, which uses the ground sampling distance equation to convert pixel measurements to cm^37,40,154^. Measurements of body length and widths for each whale were used to scale a 3D model of a humpback whale to estimate the total body volume of each individual^155^. We examined variability in humpback whale BCI between years and assessed differences BCI between 2024 and prior years using a Wilcoxon test.

### Analyses of right and sei whale aerial survey data

We assessed monthly relative abundance of right and sei whales in the NYB as the number of right or sei whales, respectively, observed per kilometer of effort for all months with at least 200 km of survey effort, and examined how monthly relative abundance in 2024 compared to that of prior years. While studies focused on modeling marine mammal density (individuals per km^2^) must model detection distances and account for factors influencing detection probabilities^68^, here we focus on the number of right and sei whales per kilometer as a broad metric of abundance. In addition to interannual comparisons, we examined patterns in the timing and location of right and sei whale occurrence in the NYB in 2024 relative to the progression of the fresh pulse.

### Analyses of seabird survey data

We assess the number of seabirds per km of survey effort as a metric of relative abundance. We used a similar analytical approach as for aragonite and *C. finmarchicus* data, comparing the relative abundance of seabirds in 2024 to prior years using Wilcoxon tests, correcting for multiple comparisons using Bonferroni corrections (alpha value of 0.05/4 to account for comparisons across the four seasons).

## Supplementary Information

Below is the link to the electronic supplementary material.

Supplementary Material 1

## Data Availability

Data used in this study are either available online in the data sources indicated in the Methods, Supplementary Material or papers cited, or are available on Dryad (DOI: 10.5061/dryad.3j9kd520z). For right whale aerial survey data, data can be requested via the North Atlantic right whale consortium data requests (https://www.narwc.org/narwc-databases.html).
