# Supplementary material for: Widespread ecosystem effects of an extreme fresh pulse event in a temperate marine ecosystem
Source: Sci Rep. 2026 Jun 28;16:23637. doi: 10.1038/s41598-026-57921-4 (PMC13424138; doi:10.1038/s41598-026-57921-4)

**Supplemental Materials**

**Supplementary Table 1:** Mean monthly surface salinity and surface salinity anomaly in the New York Bight by month from September 2023 through December 2024. Surface salinity anomaly is calculated relative to the 1993-2023 mean.

| **Month** | **Surface salinity** | **Surface salinity anomaly** |
| --- | --- | --- |
| Sep-23 | 32.45 | -0.10 |
| Oct-23 | 32.29 | -0.61 |
| Nov-23 | 31.92 | -1.03 |
| Dec-23 | 31.94 | -1.06 |
| Jan-24 | 31.94 | -1.03 |
| Feb-24 | 31.63 | -1.22 |
| Mar-24 | 31.37 | -1.26 |
| Apr-24 | 31.18 | -0.98 |
| May-24 | 31.03 | -0.80 |
| Jun-24 | 30.16 | -1.43 |
| Jul-24 | 30.47 | -1.13 |
| Aug-24 | 30.88 | -1.07 |
| Sep-24 | 32.13 | -0.41 |
| Oct-24 | 32.72 | -0.18 |
| Nov-24 | 33.10 | 0.15 |
| Dec-24 | 33.16 | 0.17 |

**Supplementary Table 2**: p-values for wilcoxon test results examining differences in aragonite saturation state, *Calanus finmarchicus* density, humpback whale body condition and seabird abundance between 2024 and prior years. For aragonite and *C. finmarchicus*, separate tests were done for each season and region given observed variability in the data and for aragonite saturation state, separate tests were done for surface and bottom waters, respectively. Significant values are shown in bold after correcting for multiple comparisons.

| **Aragonite saturation** |  |  |
| --- | --- | --- |
| Spring | Bottom | Surface |
| Inshore | 3.62 x 10^-2^ | **1.36 x 10^-2^** |
| Shelf | **3.46 x 10^-5^** | **5.81 x 10^-7^** |
| Offshore | 0.17 | **8.33 x 10^-6^** |
|  |  |  |
| Summer | Bottom | Surface |
| Inshore | 0.13 | 0.41 |
| Shelf | 2.52 x 10^-2^ | 0.20 |
| Offshore | 0.87 | 0.62 |
|  |  |  |
| ***Calanus finmarchicus* density** |  |  |
| Spring |  |  |
| Inshore | 0.97 | |
| Shelf | **1.53 x 10^-5^** | |
| Offshore | **1.23 x 10^-3^** | |
| Summer |  |  |
| Inshore | 0.2455 | |
| Shelf | **1.96 x 10^-5^** | |
| Offshore | 9.76 x 10^-2^ | |
|  |  |  |
| **Humpback Body Condition** | **1.87 x 10^-8^** | |
|  |  | |
| **Seabird abundance** |  | |
| Spring | 3.6 x 10^-2^ | |
| Summer | **7.8 x 10^-3^** | |
| Fall | 0.21 | |
| Winter | 6.6 x 10^-2^ | |

**Supplementary Table 3:** Fish and invertebrate species assessed from 1993-2024. Columns indicate whether a significant poleward shift was observed from 1993-2023, whether an equatorward shift was observed in 2024 (representing a reversal of recent poleward shifts for many species), and whether the equatorward shift observed in 2024 was the greatest equatorward shift observed from 1993-2024.

| **Common name** | **Species name** | **Poleward shift (1993-2023)** | **Equatorward shift in 2024** | **Greatest equatorward shift occurred in 2024** | **Equatorward shift > 200 km in 2024** |
| --- | --- | --- | --- | --- | --- |
| Spiny dogfish | *Squalus acanthias* | Y | Y | Y | Y |
| Winter skate | *Leucoraja ocellata* | Y | Y | Y | N |
| Little skate | *Leucoraja erinacea* | N | Y | Y | N |
| Atlantic herring | *Clupea harengus* | Y | Y | Y | N |
| Alewife | *Alosa pseudoharengus* | Y | Y | Y | Y |
| Blueback herring | *Alosa aestivalis* | Y | Y | Y | N |
| Silver hake | *Merluccius bilinearis* | Y | Y | Y | N |
| Red hake | *Urophycis chuss* | Y | Y | N | N |
| Spotted hake | *Urophycis regia* | Y | Y | Y | Y |
| Summer flounder | *Paralichthys dentatus* | N | Y | Y | Y |
| Fourspot flounder | *Hippoglossina oblonga* | Y | Y | Y | N |
| Winter flounder | *Pseudopleuronectes americanus* | Y | Y | N | N |
| Windowpane | *Scophthalmus aquosus* | N | Y | Y | N |
| Gulf Stream flounder | *Citharichthys arctifrons* | N | Y | Y | Y |
| Atlantic mackerel | *Scomber scombrus* | Y | Y | Y | N |
| Butterfish | *Peprilus triacanthus* | N | Y | Y | Y |
| Black sea bass | *Centropristis striata* | Y | Y | Y | Y |
| Northern searobin | *Prionotus carolinus* | Y | Y | Y | N |
| Ocean pout | *Macrozoarces americanus* | Y | Y | N | N |
| Goosefish | *Lophius americanus* | N | Y | Y | Y |
| Sea scallop | *Placopecten magellanicus* | Y | Y | N | N |
| Northern shortfin squid | *Illex illecebrosus* | N | Y | Y | Y |
| Longfin squid | *Loligo pealeii* | Y | Y | Y | Y |

**Supplementary Table 4**: Seabird species observed by year and season in the New York Bight. Species abbreviations are as follows: BLSC, black scoter (*Melanitta americana*); COLO, common loon (*Gavia immer*); COMU, common murre (*Uria aalge*); COSH, Cory’s shearwater (*Calonectris borealis*); COTE, common tern (*Sterna hirundo*); DCCO, double-crested cormorant (*Phalacrocorax auritus*); DOVE, dovekie (*Alle alle*), FOTE, Forster's tern (*Sterna forsteri*); GBBG, great black-backed gull (*Larus marinus*); GRSH, great shearwater (*Ardenna gravis*); HERG, herring gull (*Larus argentatus* *smithsonianus*); LAGU, laughing gull (*Leucophaeus atricilla*); LESP, Leach’s storm petrel (*Oceanodroma leucorhoa*); LTDU, long-tailed duck (*Clangula hyemalis*), MASH, manx shearwater (*Puffinus puffinus*); NOFU northern fulmar (*Fulmarus glacialis*); NOGA, northern gannet (*Morus bassanus*); OSPR, osprey (*Pandion haliaetus*); RAZO, razorbill (*Alca torda*); SOSH, sooty shearwater (*Ardenna griseus*); WFSP, white-faced storm petrel (*Pelagodroma marina*); WISP, Wilson's storm petrel (*Oceanites oceanicus*); WWSC, white-winged scoter (*Melanitta fusca*)

| **Season** | **Dates** | **Transects completed** | **Species** |
| --- | --- | --- | --- |
| Spring 2023 | 3/27, 5/10 & 5/11 | 1-4 | BLSC, COLO, COSH, DCCO, DOVE, GBBG, HERG, NOGA, RAZO, WISP, WWSC |
| Spring 2024 | 4/26, 4/29 | 1-4 | COLO, COMU, DCCO, FOTE, GBBG, HERG, LAGU, NOGA, RAZO, SOSH, WISP |
| Spring 2025 | 5/20 | 1,2 | COLO, COTE, GBBG, GRSH, HERG, NOGA |
|  |  |  |  |
| Summer 2022 | 8/22 & 8/23 | 1-4 | COTE, COSH, GBBG, GRSH, HERG, LAGU, SOSH, WFSP, WISP |
| Summer 2023 | 7/25 & 7/26 | 1-4 | COTE, COSH, GBBG, GRSH, HERG, LAGU, OSPR, WISP |
| Summer 2024 | 6/11, 7/9, 8/13, 8/14 | 1-4 | COLO, COTE, COSH, GBBG, GRSH, HERG, LAGU, LESP, NOGA, OSPR, SOSH, WFSP, WISP |
| Summer 2025 | 6/13 | 3,4 | COLO, COSH, COTE, GBBG, GRSH, HERG, SOSH, WISP |
|  |  |  |  |
| Fall 2022 | 11/1, 11/2, 11/15 | 1-4 | BLSC, COSH, DCCO, DOVE, GBBG, HERG, LAGU, NOFU, NOGA |
| Fall 2023 | 10/24, 10/26 | 1-3 | BLSC, COLO, COSH, GBBG, GRSH, HERG, LAGU, NOGA |
| Fall 2024 | 10/21, 10/22 | 1-4 | COSH, DCCO, GBBG, GRSH, HERG, LAGU, MASH, NOGA |
| Fall 2025 | 9/8, 11/29 | 1,3,4 | BLSC, COLO, COMU, COSH, GBBG, GRSH, HERG, LAGU, NOGA, WFSP, WISP, WWSC |
|  |  |  |  |
| Winter 2022-2023 | 1/30/2023 | 3,4 | GBBG, HERG, LTDU, NOGA, UNAU, UNBI, UNGU, UNSCOTER, WWSC |
| Winter 2023-2024 | 12/8/2023 | 3,4 | COLO, DOVE, HERG, LAGU, NOGA, UNBI, UNGU, UNSCOTER, UNTERN |
| Winter 2024-2025 | 1/17/2025 | 1,2 | COLO, COMU, DCCO, DOVE, GBBG, HERG, LAGU, NOGA, UNBI, UNGU, UNTERN, WWSC |

**Supplementary Table 5**: Number of samples for carbonate chemistry analyses by year, season and depth bin.

| Year | Season | Depth grouping | | | | | |
| --- | --- | --- | --- | --- | --- | --- | --- |
|  |  | <35m | | 35-70m | | >70m | |
|  |  | Surface | Bottom | Surface | Bottom | Surface | Bottom |
| 2019 | Spring | 0 | 2 | 1 | 3 | 4 | 6 |
| 2019 | Summer | 5 | 4 | 12 | 11 | 11 | 27 |
| 2020 | Summer | 6 | 3 | 4 | 6 | 0 | 0 |
| 2021 | Spring | 8 | 6 | 16 | 24 | 8 | 22 |
| 2022 | Spring | 1 | 1 | 3 | 2 | 3 | 1 |
| 2022 | Summer | 7 | 6 | 13 | 12 | 10 | 9 |
| 2023 | Spring | 4 | 4 | 12 | 12 | 14 | 12 |
| 2024 | Spring | 5 | 3 | 13 | 10 | 12 | 12 |
| 2024 | Summer | 2 | 2 | 6 | 5 | 4 | 4 |

**Supplementary Table 6**: Number of samples for zooplankton analyses by year, season and depth bin.

| Year | Season | Depth grouping | | |
| --- | --- | --- | --- | --- |
|  |  | <35m | 35-70m | >70m |
| 2018 | Summer | 2 | 4 | 3 |
| 2019 | Spring | 1 | 1 | 5 |
| 2019 | Summer | 1 | 10 | 2 |
| 2020 | Summer | 2 | 4 | 0 |
| 2021 | Spring | 11 | 31 | 21 |
| 2022 | Spring | 1 | 4 | 6 |
| 2022 | Summer | 9 | 14 | 5 |
| 2023 | Spring | 5 | 11 | 12 |
| 2024 | Spring | 4 | 12 | 11 |
| 2024 | Summer | 5 | 10 | 5 |

**Supplementary Figure 1:** Monthly sea surface temperature anomaly from September 2023 through December 2024 in the New York Bight (data from NOAA OISST). Anomalies were calculated relative to the 2010-2023 monthly means.


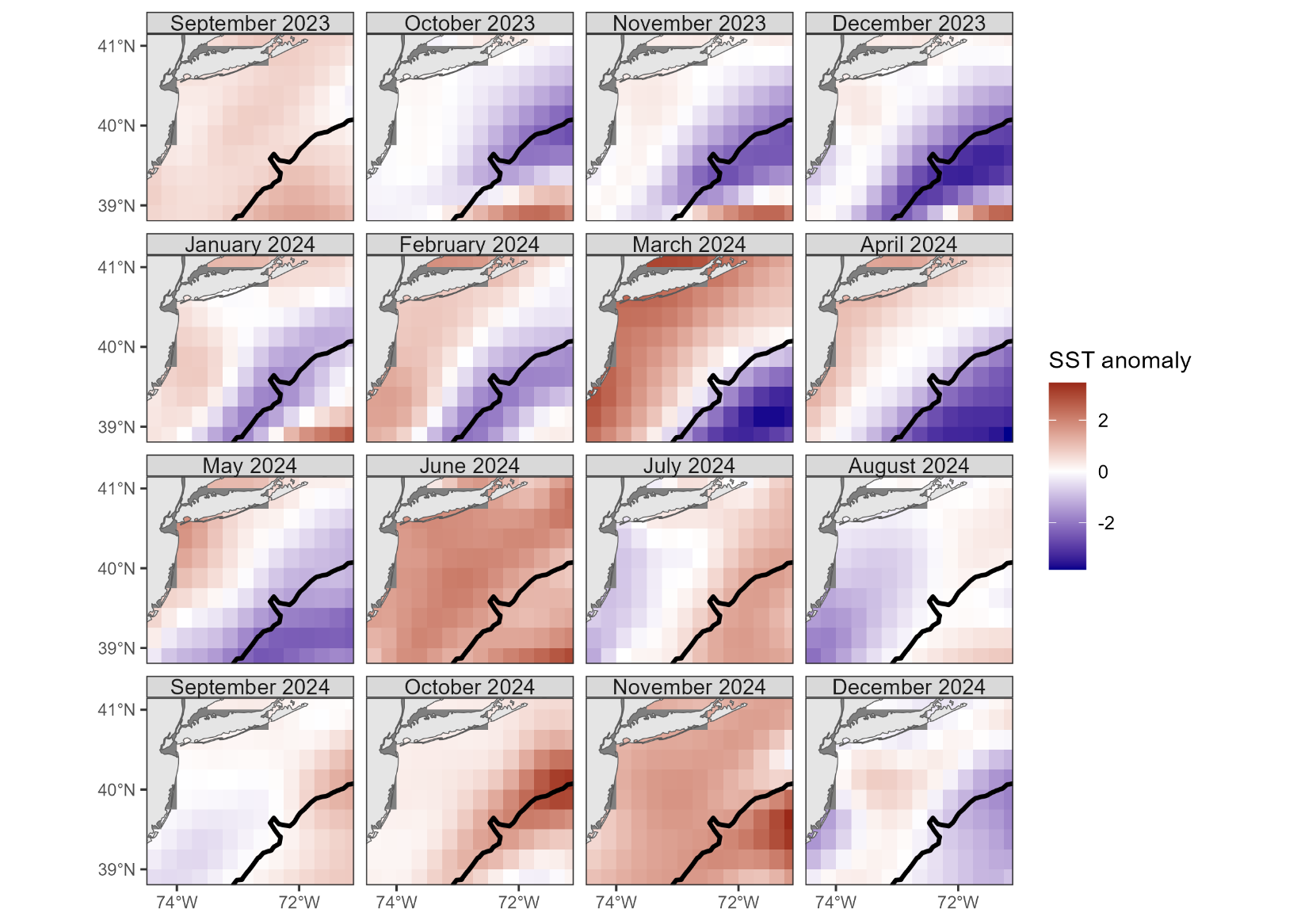


**Supplementary Figure 2:** Seasonal variability in temperature and salinity data from cross-shelf glider transects in 2024 (purple and blue) in comparison to 2019-2023 (grey).


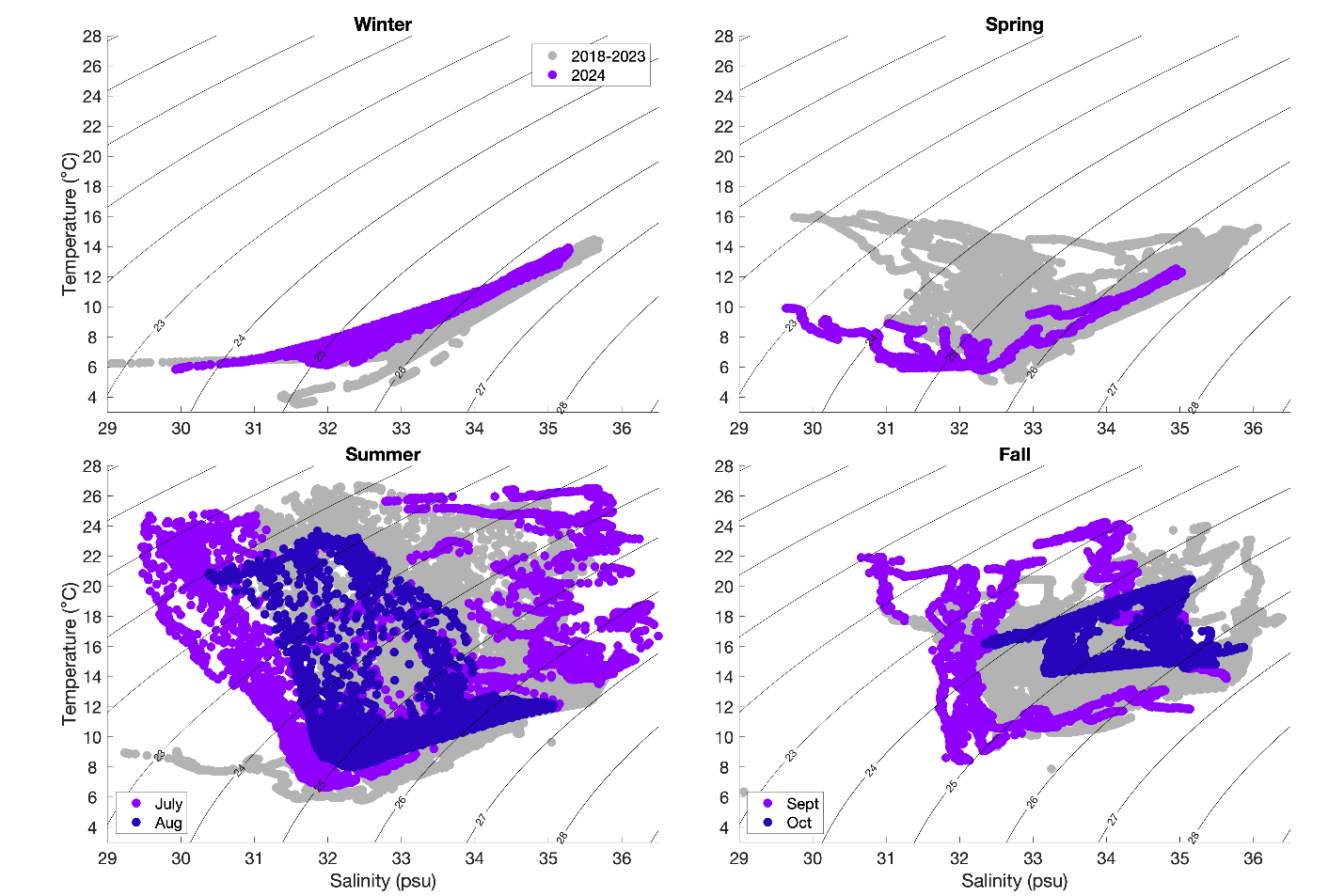


**Supplementary Figure 3**: Interpolated sea surface salinity data from summer research cruises in the New York Bight in 2018, 2019, 2020, 2022 and 2024. Colored circles represent measured values from surface CTD data, while continuous surfaces represent interpolations between CTD stations. Cruises were conducted in July 2018, July 2022 and 2024, in May 2019, and in June 2020. Summer cruises were not conducted in 2021 or 2023.


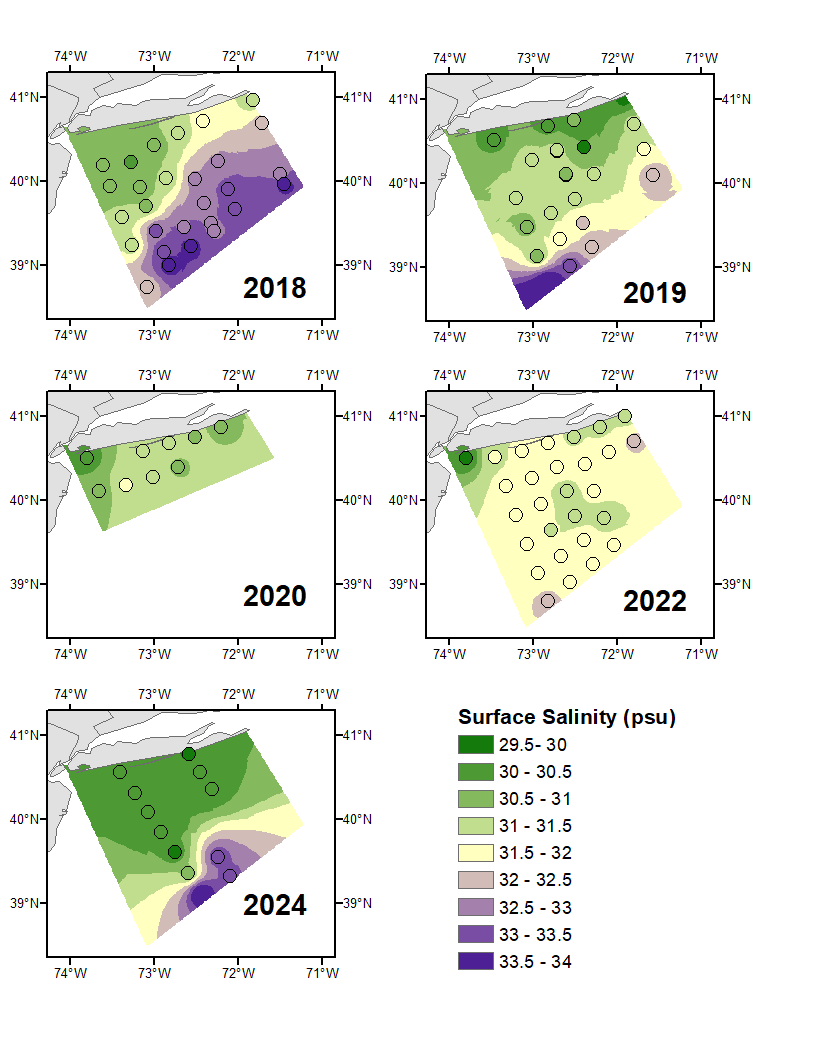


**Supplementary Figure 4**: Annual total precipitation (mm) measured at the meteorological station at Islip Airport plotted against the New York Bight regional-averaged, annual- averaged surface salinity (psu) from satellite. There was no significant linear relationship between average annual salinity in the New York Bight and annual total precipitation.


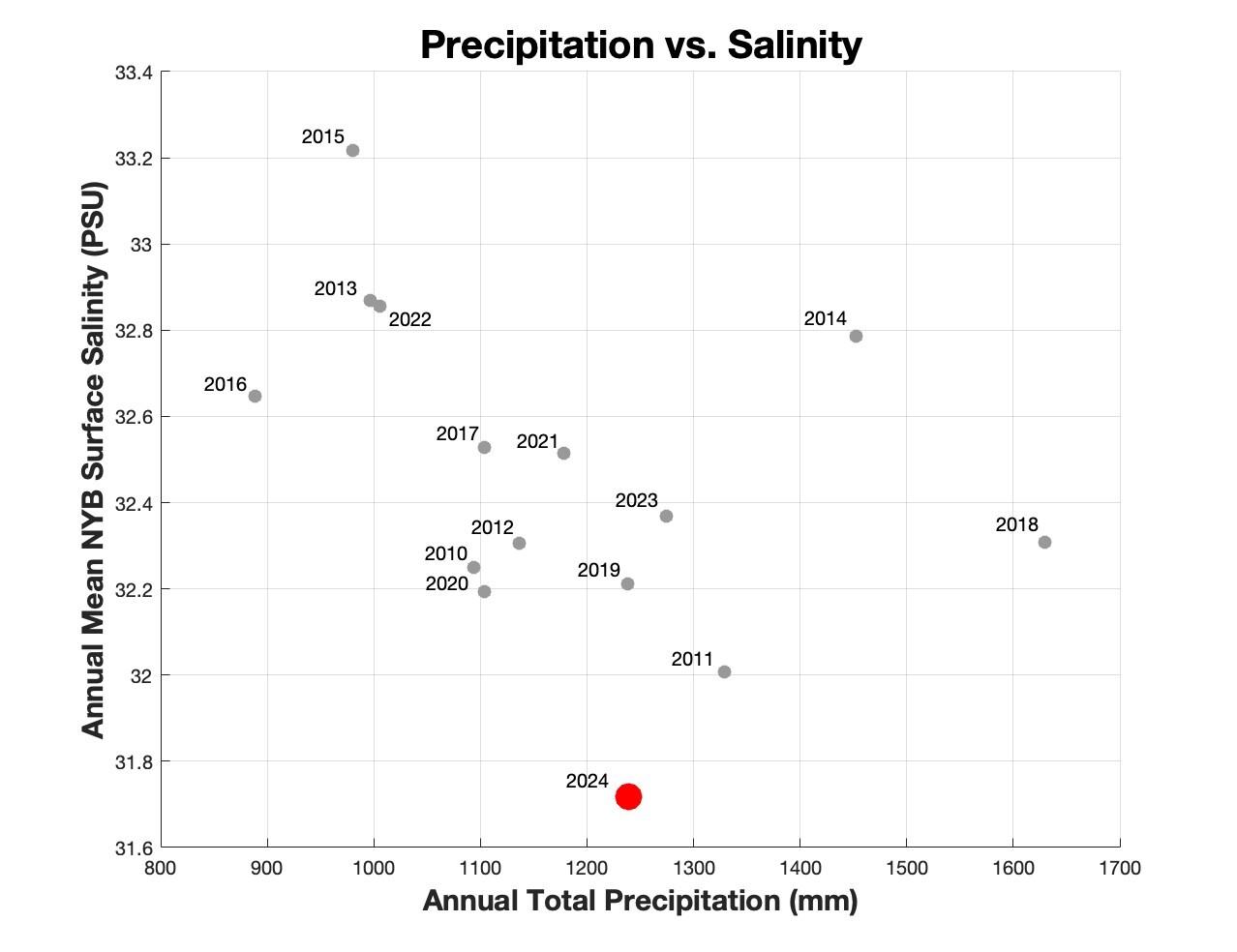


**Supplementary Figure 5**: Surface aragonite saturation state by season (spring and summer) and depth bin from 2019-2024.


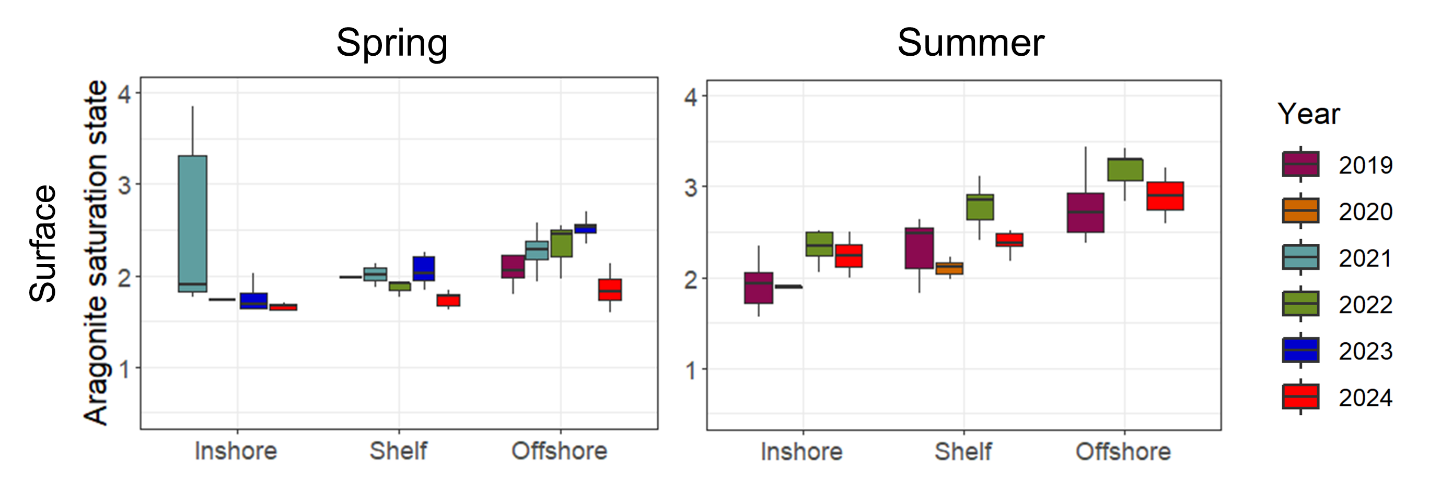


**Supplementary Figure 6**: Seasonal mean abundances of *Calanus finmarchicus* in the New York Bight from 1993-2021 in inshore, shelf and offshore waters, produced using ECOMON data. Outliers are not shown here as extreme values skew the y axis and make it difficult to observe differences between inshore, shelf, and offshore regions.


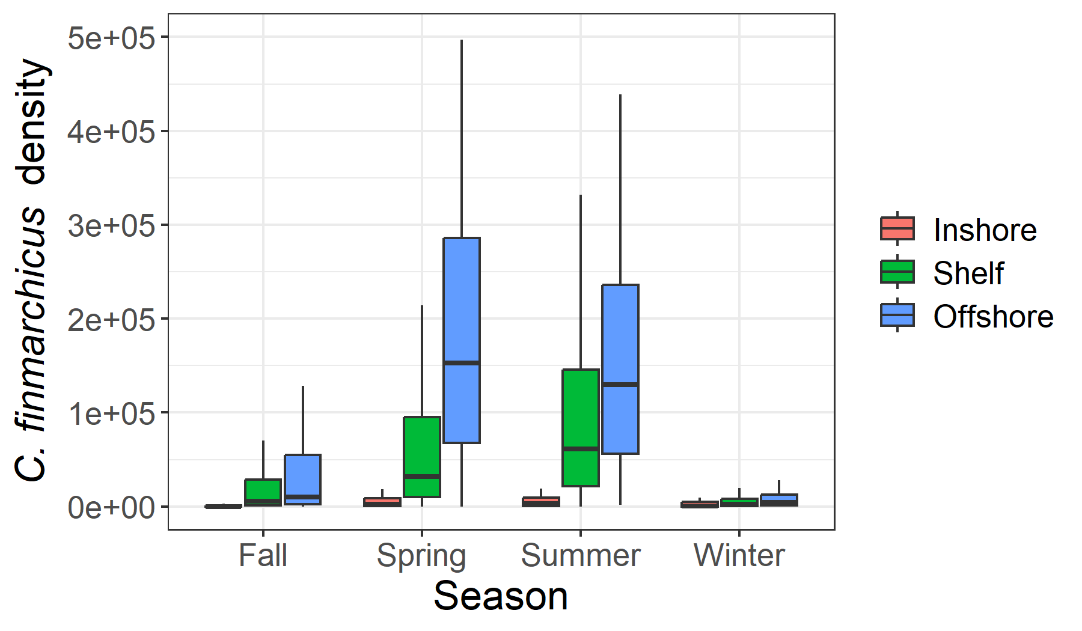


**Supplementary Figure 7**: Estimated residence time and number of humpback whales in the eastern New York Bight by year from photo-identification analyses


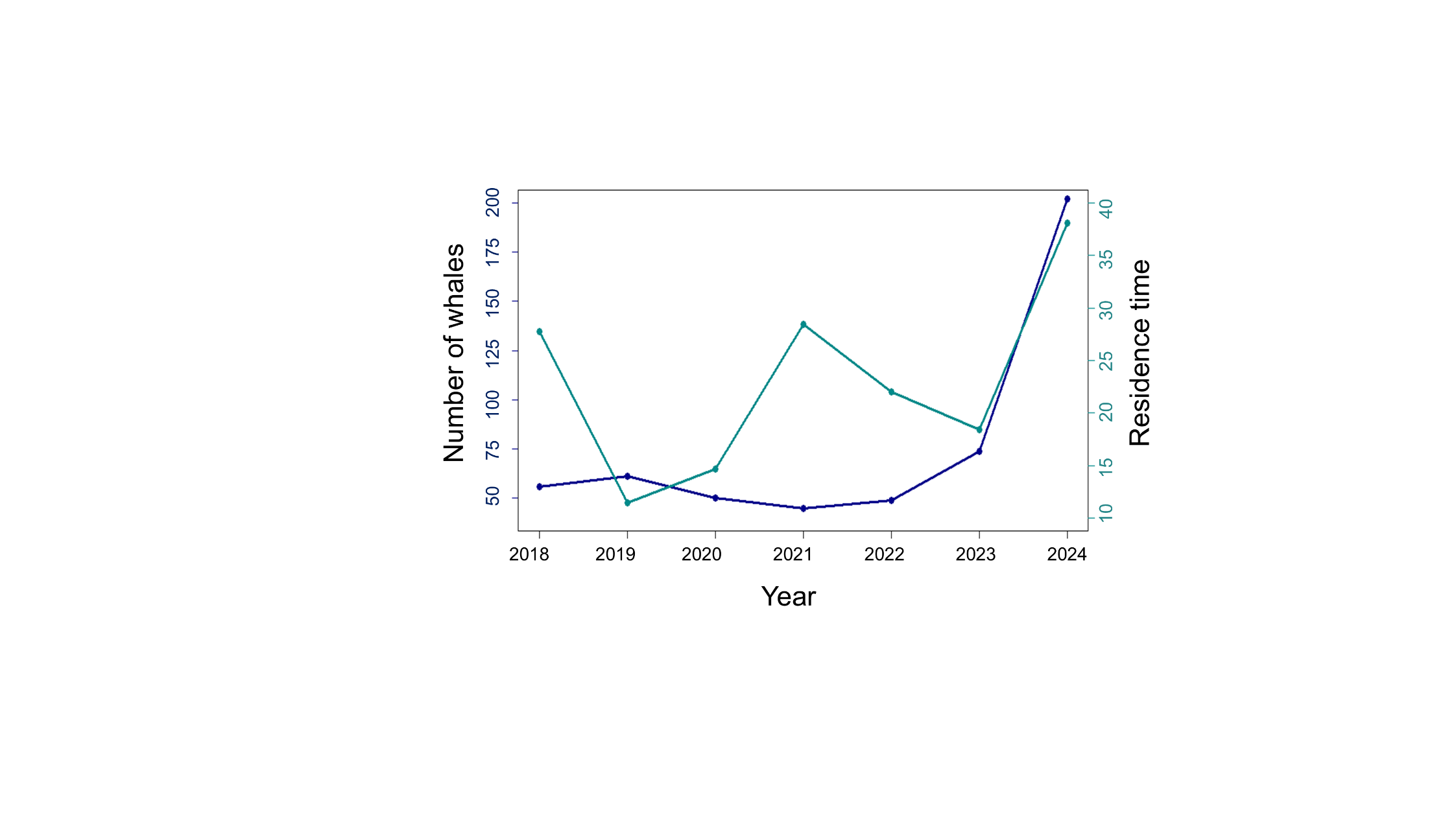


**Supplementary Figure 8:** Boxplots showing whales per kilometer of survey effort for North Atlantic right whales (top) and sei whales (bottom panel) from available data in 2001-2023 (grey boxplots) relative to 2024 (red dots for right whales, yellow dots for sei whales). Grey dots represent outliers during 2001-2023. For right whales in all months and sei whales in all months but May and July, boxplots showing data from years prior to 2024 are not visually evident as the number of whales per km is much higher in 2024.


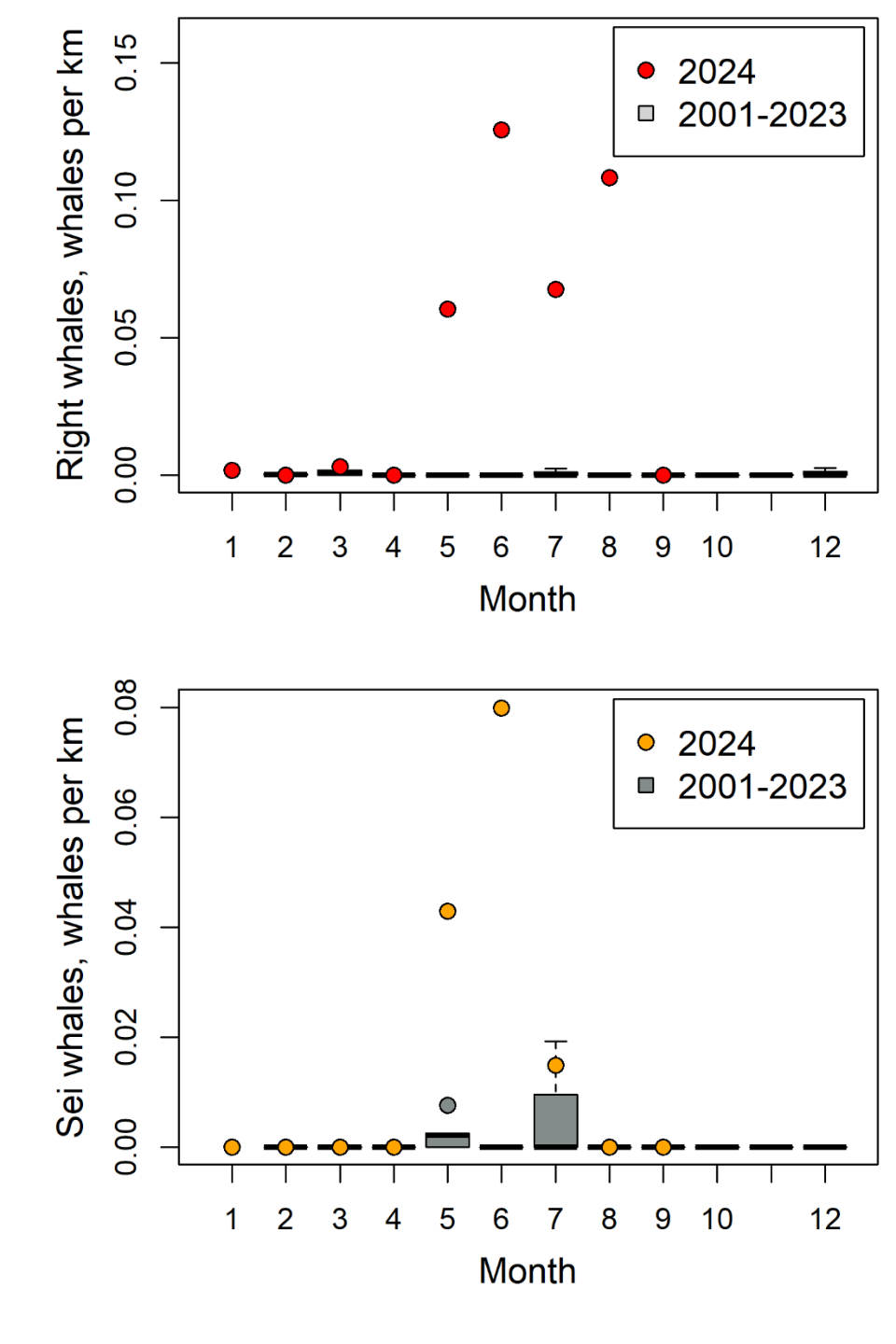


**Supplementary Figure 9** (a) Right whale sightings and survey effort in 2024 and (b) in 2001-2023. Due to the increased survey effort in the Hudson Canyon region in the southwest region of the New York Bight study area in 2024 (entire study area shown in green), we separately assessed sightings per unit effort by excluding this region for comparison (modified study area excluding the southwest region in blue). Monthly average whales per km survey effort for right whales (c) and sei whales (d) remained elevated in July when the southwest region of the study area was omitted. Surveys in the New York Bight in May and June 2024 primarily occurred in the southwestern region of the New York Bight (<200 km surveyed outside of this region). Thus, when excluding this region, values of whales per km were not available in May June 2024 which showed some the highest values for sei whales when the full New York Bight region was assessed.


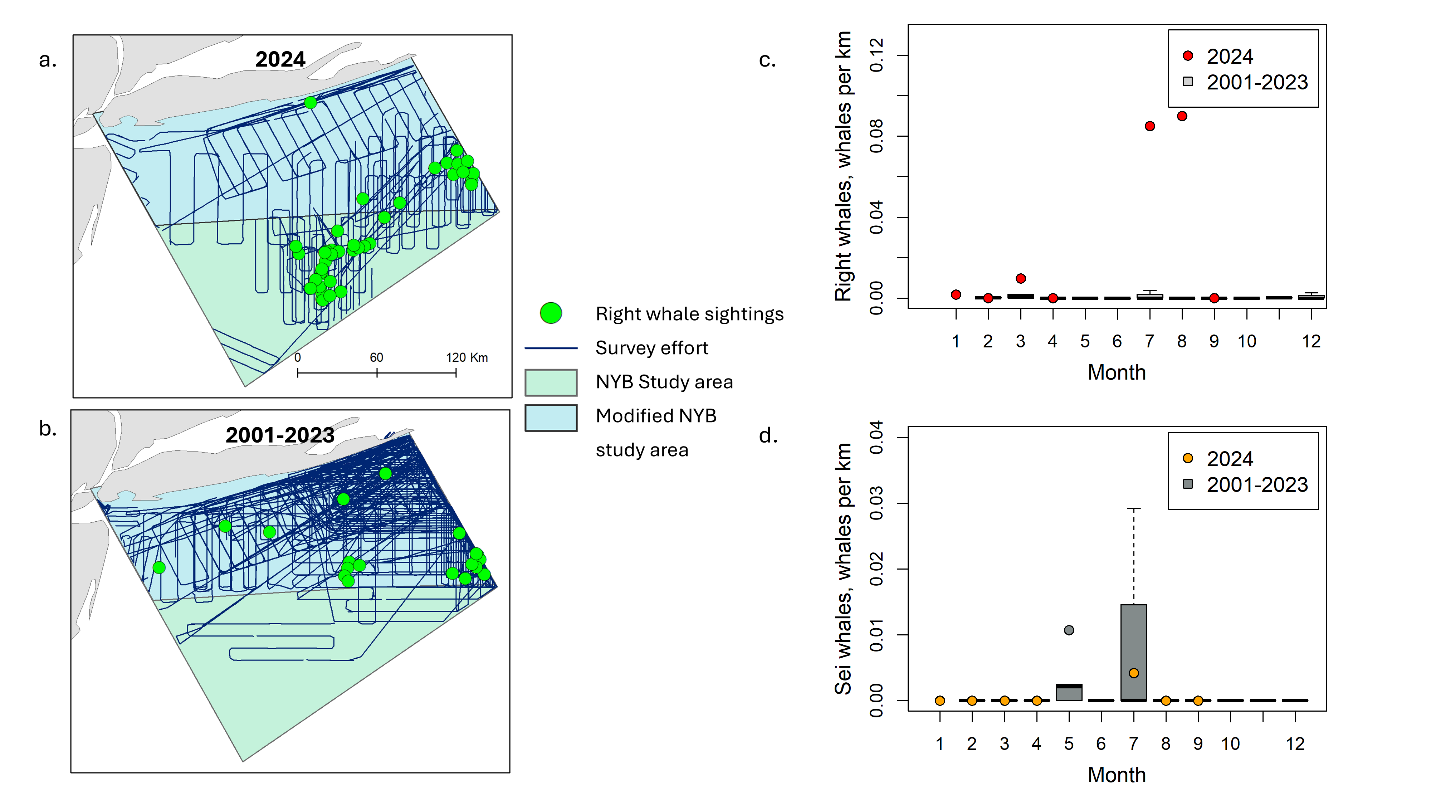


**Supplemental Figure 10:** Results of seabird surveys conducted in the New York Bight. a. Number of seabirds observed per kilometer of survey effort in spring, summer and fall 2022-2025. b. Species accumulation curves for seabirds observed by season.

**
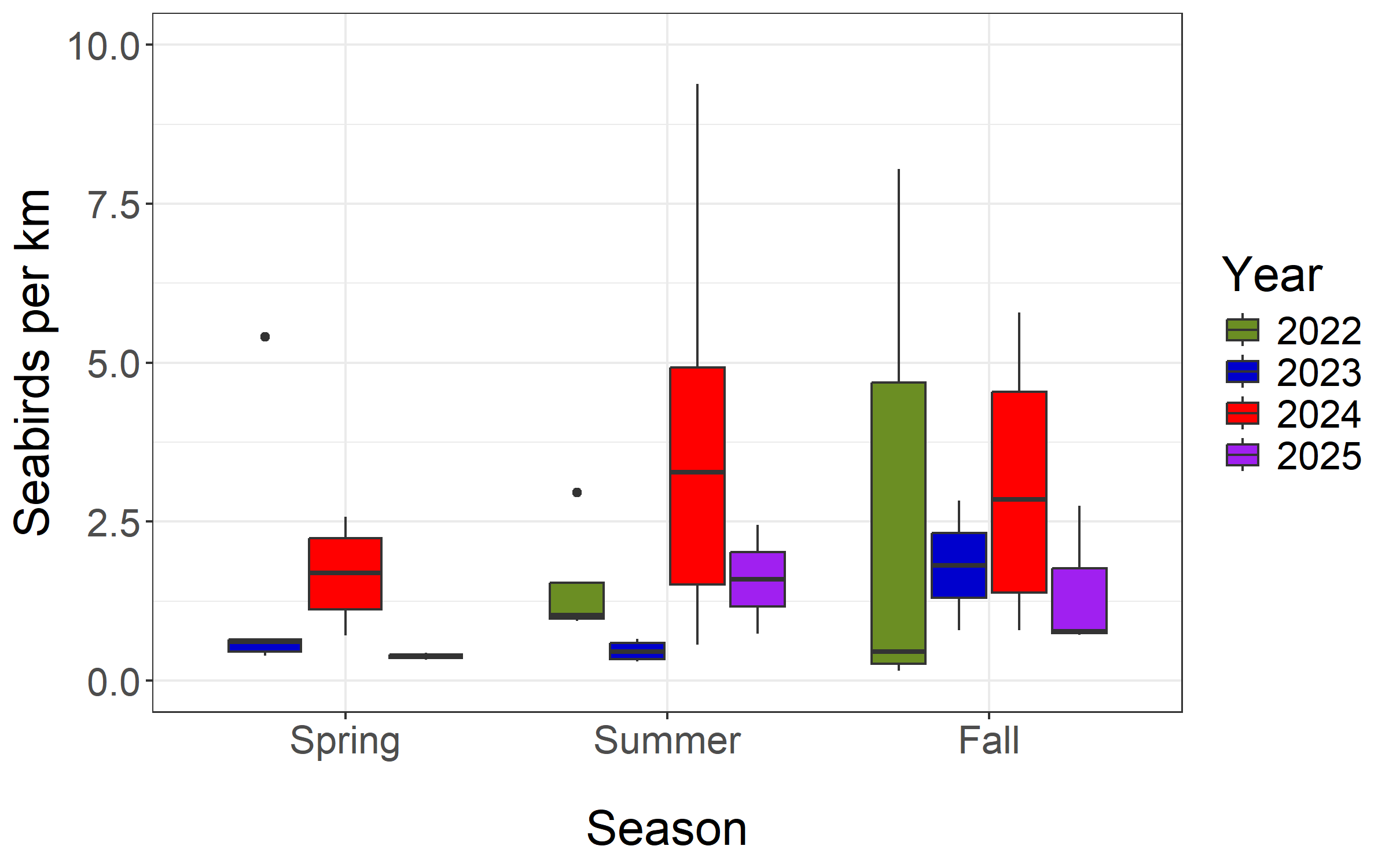
**

**Supplemental Figure 11:** a. Ship-based survey transects used for zooplankton tows, CTD casts and carbonate chemistry samples (NYOS Transects) and Seabird survey transects in the New York Bight. b. Glider transects used to assess cross-shelf profiles of temperature and salinity. c. Location of humpback whale sightings used for photo-identification studies. Red extent indicator in panel a shows the extent of panels b and c.


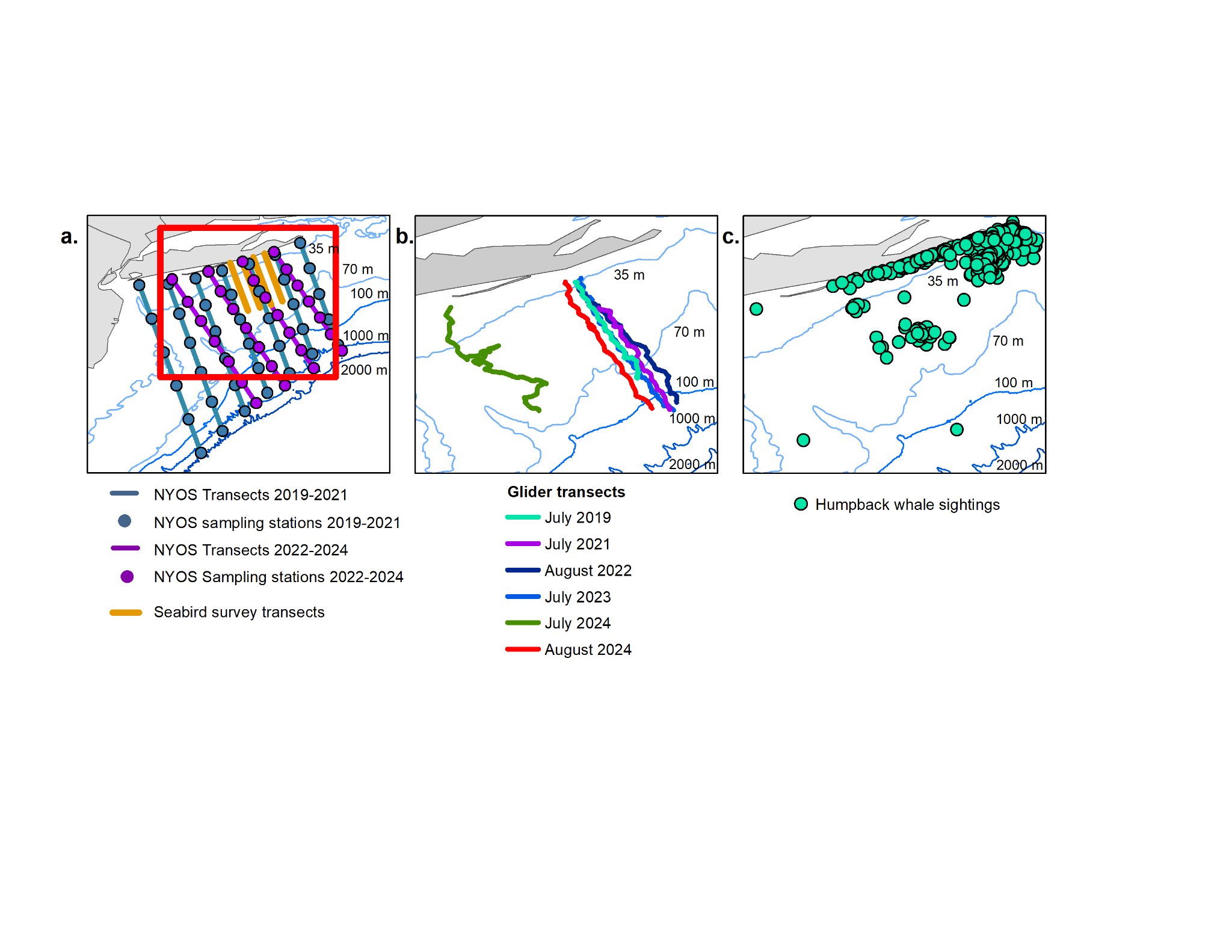

Supplement: Supplementary file 1 — Supplementary Material 1 [file 41598_2026_57921_MOESM1_ESM.docx]
